# The *PagWUS-PagCLV3* module regulates shoot meristem maintenance and activity in poplar

**DOI:** 10.48130/forres-0026-0007

**Published:** 2026-03-26

**Authors:** Wan Chen Dong, Fang Li Wang, Xiao Tong Li, Xiu Xia Wu, Li Fang Yu, Lin Lin Luan, Yan Wang, Xian Sheng Zhang, Zhi Juan Cheng, Ya Lin Sang

**Affiliations:** College of Forestry, College of Life Sciences, Shandong Agricultural University, Tai’an, Shandong 271018, China

**Keywords:** *CLV3*, Poplar, Shoot meristem, Shoot regeneration, *WUS*

## Abstract

The shoot apical meristem of vascular plants generates all the aboveground organs. During this process, the structure and function of the meristem are maintained by a group of regulatory genes, among which the *WUSCHEL* (*WUS*)-*CLAVATA3* (*CLV3*) module plays the core role. To date, all of the insights into shoot meristem homeostasis have been derived from studies on herbaceous plants. The mechanism by which the shoot meristem is maintained in trees remains unknown. In this study, we analyzed the functions of the poplar genes *PagWUS* and *PagCLV3*, homologs of *Arabidopsis* *WUS* and *CLV3*, respectively, in the maintenance and regeneration of the shoot meristem. Our results reveal both conserved and divergent functions compared to those of their orthologs in herbaceous species. Similar to their herbaceous counterparts, *PagWUS* and *PagCLV3* are specifically expressed in the organizing center and stem cells, respectively, and form a feedback loop that regulates shoot meristem maintenance. Overexpression of *PagWUS* promoted shoot regeneration. Compared with herbaceous species, poplar possesses a much larger stem cell niche. The function of the *PagWUS-PagCLV3* module is consistent with the developmental characteristics of perennial trees in that it regulates the cessation of the shoot meristem and mediates the proper pattern of secondary growth. Disruption of *PagCLV3* enhanced shoot regeneration capacity. Our results shed light on shoot meristem regulation in trees and pave the way for understanding the mechanisms of meristem activity and plant development.

## Introduction

Unlike most animals, vascular plants exhibit continuous postembryonic development, primarily driven by meristematic activity^[1]^. The shoot apical meristem produces aboveground organs, the root apical meristem generates root tissues, and both meristems contribute to the postembryonic development of vascular plants^[2]^. The shoot apical meristem, which gives rise to the aboveground parts, can be divided into three functional domains^[3]^. Located in the center and summit of the shoot meristem, the central zone (CZ) harbors a mass of pluripotent stem cells. During postembryonic development, shoot stem cells continuously divide to generate daughter cells and simultaneously maintain themselves. The daughter cells are pushed laterally into the peripheral zone (PZ) to generate lateral organs, or downwardly to form the stem. A group of specialized cells beneath the CZ comprises the organizing center (OC), which is required for maintaining the stem cells^[4]^.

The elaborate activity of the shoot meristem is controlled by a number of regulatory genes, among which the *WUSCHEL*-*CLAVATA* (*WUS*-*CLV*) module plays a central role^[5]^. *WUS* is specifically expressed in the OC and encodes a homeodomain transcription factor^[6]^. After translation, WUS migrates upward into the CZ through the plasmodesmata and activates the transcription of *CLV3*, the stem cell marker gene^[7,8]^. *CLV3* encodes a secreted peptide, which is perceived by multiple sets of receptor complexes^[9]^. CLV3 signaling restricts *WUS* expression in the OC through ligand-receptor interactions involving CLV1, CLV2, and CLV3 itself, leading to the inhibition of *WUS* transcription^[10,11]^. Defects in *WUS* result in stem cell consumption, while mutations of *CLV3* lead to over-proliferation of stem cells and enlargement of the meristem^[6,10]^. Thus, *WUS* and *CLV3* form a regulatory feedback loop that controls shoot meristem homeostasis, where WUS activates *CLV3* expression and CLV3-signaling restricts *WUS* expression.

Recent studies have revealed regulatory roles of the L1-miR171-HAM signaling cascade in maintaining the *WUS*-*CLV* module. HAIRY MERISTEMs (HAMs) are GRAS family transcription factors that form heterodimers with WUS and repress its ability to activate *CLV3* expression in the OC^[12−14]^. Meanwhile, ARABIDOPSIS THALIANA MERISTEM LAYER1 and PROTODERMAL FACTOR2 specifically activate the transcription of miR171 in the epidermal cell layer^[15]^. Mature miR171 diffuses downward and then targets and cleaves *HAMs* transcripts in the CZ, thereby allowing *CLV3* expression therein^[16]^. WUS acts in parallel with the KNOX1 family homeodomain transcription factor SHOOTMERISTEMLESS (STM)^[17]^. *STM* is expressed throughout the shoot meristem except for incipient primordia. STM stimulates cytokinin biosynthesis, suppresses differentiation, and maintains the proliferation of meristematic cells^[18,19]^. A recent study showed that STM interacts with WUS and enhances the binding of WUS to the *CLV3* promoter, demonstrating the coordination between STM and the *WUS*-*CLV* module^[20]^.

The phytohormones cytokinin and auxin play important roles in regulating the homeostasis of the shoot meristem. The cytokinin biosynthetic gene *LONELY GUY4* is expressed in the epidermis of the shoot meristem and probably establishes a cytokinin gradient that extends into the OC, where the expression of *AHK4* (encoding the cytokinin receptor ARABIDOPSIS HISTIDINE KINASES 4) overlaps with that of *WUS*^[21,22]^. Type-B ARABIDOPSIS RESPONSE REGULATORs (ARRs), the key regulators of cytokinin response genes, directly activate *WUS* transcription and sustain its expression level *via* suppressing auxin biosynthesis in the OC^[22−26]^. Cytokinin signaling is required for stabilizing the WUS protein^[27]^. WUS in turn represses the expression of type-A ARRs, which encode cytokinin signaling repressors^[28]^. Therefore, cytokinin signaling and *WUS* expression form a positive feedback loop to confine the appropriate organization of the shoot meristem. Auxin signaling is functionally connected to stem cell behavior^[29]^. In the CZ, *WUS* rheostatically controls auxin signaling *via* regulating histone acetylation at target loci, including genes involved in the auxin pathway. This permits low levels of auxin signaling, which enables stem cells to be resistant to auxin-induced differentiation, and is required for their maintenance^[30]^. Another study has shown that AUXIN RESPONSE FACTOR 5 mediates auxin signaling in the CZ by directly repressing the transcription of *DORNROSCHEN*, which encodes a positive regulator of *CLV3* expression^[31]^. The findings of those studies revealed the molecular mechanism of auxin-controlled stem cell homeostasis.

Under proper *in vitro* culture conditions, the shoot meristem can be regenerated from somatic tissues^[32]^. When detached explants are cultured on auxin-rich callus induction medium, they produce callus, a mass of proliferating cells with transcriptional similarities to lateral root primordia but lacking a fully defined meristem identity^[33]^. After transfer onto cytokinin-rich shoot induction medium, type-B ARRs initiate *WUS* expression in clusters of callus cells and subsequently reestablish shoot meristems^[23]^. This process enables regeneration of the entire plant body and provides the essential foundation for *in vitro* propagation, genetic transformation, and the generation of virus-free plants^[34,35]^. Several lines of evidence indicate that *WUS* is essential for the regeneration of the shoot meristem^[23,25,36]^_._

Despite their similar structural features, shoot meristems of annual and perennial species exhibit distinct activities^[37]^. Annual plants preserve shoot meristem activity throughout the growth season and complete their life cycle before winter. However, for perennial plants such as trees in temperate and boreal regions, the shoot meristem perceives photoperiod and/or temperature signals and ceases activity in autumn^[38]^. Subsequently, bud scales form to protect the meristem from freezing injury^[39]^. As winter progresses, shoot meristems are gradually released from dormancy, resume activity, and give rise to bud flush^[40]^. In poplar, the induction of dormancy is controlled by the integration of photoperiod perception and the *FLOWERING LOCUS T* (*FT*) module^[41]^. Light signals are detected by phytochrome receptors and circadian clock components^[40]^. The circadian components LATE ELONGATED HYPOCOTYL 1 (LHY1), LHY2, GIGANTEA, and CONSTANS regulate the expression of *FT2* in response to day length^[42−45]^. Downregulation of *FT2* induces dormancy, while its overexpression prevents growth cessation and bud set^[41]^.

Perennial woody plants account for 42.7% of angiosperms and represent a large proportion of global biodiversity^[46,47]^. Seasonal dormancy and secondary growth are typical characteristics of woody species. Signal transduction that induces meristem cessation has been studied in detail^[48]^. However, there is no link between cessation signaling and shoot meristem activity. Whether the maintenance of the shoot meristem affects secondary growth remains unknown. In this study, we investigated the regulatory roles of the poplar genes *PagWUS* and *PagCLV3*, orthologs of Arabidopsis *WUS* and *CLV3,* respectively. The results reveal their conserved as well as distinct functions compared with those of their orthologs in herbaceous species, consistent with the developmental characteristics of perennial trees.

## Materials and methods

### Plant materials and growth conditions

Poplar (*Populus alba* × *Populus glandulosa* var. *glandulosa*) clone 84 K was used as the wild-type background. The leaves of sterile saplings cultured on rooting medium for 4−6 weeks were used as explants for genetic transformation. Phenotypic and histological analyses were performed using sterile saplings cultured on rooting medium for 2 months. Saplings grown on rooting medium for 2 months and transferred to soil for 1 month were used for meristem cessation analysis. Poplar saplings were grown at 25 °C, and Arabidopsis (*Arabidopsis thaliana*) seedlings at 22 °C. The tobacco (*Nicotiana benthamiana*) plants used for subcellular localization analysis were grown at 25 °C. All three types of plant materials were grown under a 16-h light/8-h dark photoperiod. The Arabidopsis *wus-1* and *clv3-2* mutants were prepared as described previously^[34,49]^.

### *In situ* hybridization

Shoot tips, internodes, and leaf tips derived from sterile saplings cultured on rooting medium for 2 months were fixed in FAA (alcohol : formaldehyde : acetic acid : water, 10:2:1:7) at 4 °C overnight. The material was then dehydrated and embedded in Paraplast (Sigma-Aldrich). The full-length coding region containing a digaoxin label was synthesized *in vitro*, and used as a hybridization probe as previously described^[23]^. The sections with hybridization signals were viewed and imaged under an Olympus BX-51 microscope (Olympus, Tokyo, Japan).

### Comparison of the stem cell niche area

The height, width, and area of *CLV3*/*WUS* expression domains, as well as the number of cells within these domains, were measured from the *in situ* hybridization images using ImageJ software as previously described^[50,51]^. The shoot meristem width was measured at the maximum width between leaf primordia.

### Plasmid construction and genetic transformation

To construct overexpression vectors, the full-length coding sequences of *PagCLV3-1*, *PagCLV3-2*, *PagWUS1,* and *PagWUS2* were amplified by PCR and cloned into the pROKII-GFP vector to obtain the *35S::PagCLV3-1-GFP*, *35S::PagCLV3-2-GFP*, *35S::PagWUS1-GFP,* and *35S::PagWUS2-GFP* vectors, respectively. CRISPR target sites were designed using the CRISPRdirect online tool (http://crispr.dbcls.jp), selecting sites on the basis of specificity, PAM sequence constraints, and minimal predicted off-target effects. The target sequence and the upstream and downstream 10-bp sequence were used as Blast queries against the poplar genome to ensure specificity. One target was designed in the upstream sequence of the open reading frame and one in the functional domain to improve the mutation efficiency. The target sequence with A or G at the 20^th^ bp upstream of NGG was preferentially selected (Supplementary Fig. S1). The target sequence was amplified to construct the gsRNA expression cassette, which was then assembled into the vector pYLCRISPR/Cas9-DN (kindly provided by Yao-Guang Liu, South China Agricultural University)^[52]^. Sequence changes in transgenic plants were identified by PCR amplification and DNA sequencing (Supplementary Fig. S1). The 4,430-bp sequence immediately upstream of the ATG start codon of *AtWUS* was used as the promoter, and was amplified and cloned into the pROKII-GFP vector. The *PagWUS1* coding sequence was cloned and inserted downstream of the *AtWUS* promoter to construct the *pAtWUS::PagWUS1-GFP* vector. The coding region of *PagCLV3-2* was amplified and cloned into the PFK-321 vector (kindly provided by Zhong Zhao, University of Science and Technology of China) by the LR reaction to construct the *pAtCLV3::PagCLV3-2* vector^[31]^.

To analyze the expression patterns of *PagWUS2* and *PagCLV3-1* at the transcriptional level, the 2,408-bp sequence immediately upstream of the ATG start codon of *PagWUS2* was amplified by PCR and cloned into the PZP211-GFP_3_ vector to generate the *pPagWUS2::GFP*_*3*_ vector (where GFP_3 _represents the concatemer of three GFP proteins). The 6,186-bp promoter sequence was used in the *pPagWUS2::GFP*_*3*_*-L* vector. The 4,707-bp sequence immediately upstream of the ATG start codon of *PagCLV3-1* was amplified by PCR and cloned into the PBI121-GUS vector to generate the *pPagCLV3-1::GUS* vector. To construct the *pPagWUS2::GFP*_*3*_ vector, the 4,707-bp promoter sequence of *PagCLV3-1* was cloned upstream of *GFP*_*3*_ in the PZP211-GFP_3_ vector, and the 1,791-bp sequence downstream of the translational stop codon was added downstream of *GFP*_*3*_. The sequences of all primers are listed in Supplementary Table S1.

The vectors were transformed into poplar *via* *Agrobacterium*-mediated transformation, using leaves as explants. The floral dip method was used for Arabidopsis transformation.

### ChIP-qPCR assay

Regenerated shoots of *pPagRR13::PagRR13-GFP* lines were used for ChIP-qPCR analyses. After harvest, 1 g tissue was cross-linked with 1% (v/v) formaldehyde under vacuum for 10 min. The cross-linking reaction was quenched with 0.125 M glycine for 5 min. The tissue was thoroughly ground in liquid nitrogen, and chromatin was extracted before sonication. Chromatin was sheared by sonication (15 min at 40% power for 10 s on/off) to generate DNA fragments between 200 bp and 1 kb. Of the DNA fragment mixture, 5% of the volume was saved as the input control. The anti-GFP antibody (TransGen Biotech, Beijing, China) was pre-incubated with Protein A/G Magnetic Beads (Selleckchem, Houston, TX, USA) at 4 °C for 2 h with gentle rotation. Chromatin was pre-cleared by incubation with the Protein A/G Magnetic Beads before adding antibody-bound beads, and then incubated overnight at 4 °C. The beads were successively washed with low-salt washing buffer (150 mM NaCl, 0.1% [w/v] SDS, 1% [v/v] Triton X-10, 2 mM EDTA [pH 8.0], and 20 mM Tris-HCl [pH 8.0]), high-salt washing buffer (500 mM NaCl, 0.1% [w/v] SDS, 1% [v/v] Triton X-10, 2 mM EDTA [pH 8.0], and 20 mM Tris-HCl [pH 8.0]), LiCl washing buffer (0.25 M LiCl, 1% [v/v] NP-40, 1% [w/v] sodium deoxycholate, 1 mM EDTA [pH 8.0], and 10 mM Tris-HCl [pH 8.0]), and TE buffer (1 mM EDTA [pH 8.0] and 10 mM Tris-HCl [pH 8.0]). Finally, the immunoprecipitated chromatin was eluted with elution buffer (1% [w/v] SDS and 0.1 M NaHCO_3_). The immunoprecipitation complex was dissociated by adding 5 M NaCl and stirring using a Thermo Mixer at 700 rpm for at least 8 h or overnight. The DNA was recovered using ChIP DNA Clean & Concentrator (Zymo Research, Irvine, CA, USA) and analyzed by qRT-PCR as described previously^[23,53]^. The sequences of all primers are listed in Supplementary Table S1.

### qRT-PCR

Total RNA was extracted from shoot tips and explants using the cetyl trimethyl ammonium bromide (CTAB) method. HiScriptII qRT Super Mix (Vazyme, Nanjing, China) was used to obtain cDNA, and 1–2 μg total RNA was used for cDNA construction. qRT-PCR was performed on a LightCycler 96 system (Roche, Basel, Switzerland) using Cham Q SYBR qPCR Master Mix (Vazyme) with gene-specific primers. Data are mean ± sd of three independent biological repeats. Data were analyzed by two-tailed Student’s t-tests, and *** indicates significant difference at *p* < 0.001. The housekeeping gene *TUBULIN* was employed as an internal reference. All primers are listed in Supplementary Table S1.

### Shoot regeneration analysis

Poplar saplings were grown under sterile conditions at 25 °C with a 16-h light/8-h photoperiod for 4–6 weeks. The 3^rd^ to 5^th^ leaves from the shoot tip were excised and cultured on differentiation medium (Murashige and Skoog medium [MS] + 30 g/L sucrose + 0.5 mg/L 6-benzyl aminopurine [6-BA] + 0.1 mg/L naphthaleneacetic acid [NAA] + 0.002 mg/L thidiazuron [TDZ] + 8 g/L agar) under light intensity of 100 μmol/m^2^/s. The number of regenerated shoots was counted after 30 d of culture. Arabidopsis seeds were germinated on ½ MS medium at 22 °C under a 16-h light/8-h dark photoperiod, and then grown for 7 d before excising cotyledons for culture. Cotyledons were cultured on callus induction medium (CIM) (MS + 0.5 g/L 2-(N-morpholino) ethane sulfonic acid hydrate [MES] + 10 g/L sucrose + 0.5 mg/L 2,4-dichlorophenoxyacetic acid [2,4-D] + 0.05 mg/L kinetin [KT] + 8 g/L agar) for 6 d before transfer onto shoot induction medium (SIM) (MS + 0.5 g/L MES + 10 g/L sucrose + 2.5 μM 2-isopentenyladenine [2-IP] + 0.9 μM indoleacetic acid [IAA] + 8 g/L agar) for shoot induction under continuous light at 100 μmol/m^2^/s. The number of regenerated shoots was counted after 21 d of culture on SIM (SIM21)^[54]^. The data are presented as mean ± sd from independent biological replicates. For each analysis, *n* > 20.

### PagCLV3-2 peptide *in vitro* treatment

The PagCLV3-2 peptide (ELRAVPSGPDPLHH) was artificially synthesized (Sangon Biotech, Shanghai, China)^[55]^. The shoots of the *CRISPR-PagCLV3-2* plants showing fasciation and phyllotaxis phenotypes were cultured on differentiation medium (MS + 30 g/L sucrose + 0.5 mg/L 6-BA + 0.1 mg/L NAA + 0.002 mg/L TDZ + 50 mg/L kanamycin + 200 mg/L timentin + 8 g/L agar) containing different concentrations of the PagCLV3-2 peptide. Complementation of the fasciation phenotype was analyzed after 30 d of culture.

### Subcellular localization analyses

The *35S::PagCLV3-1-GFP*, *35S::PagCLV3-2-GFP*, *35S::PagWUS1-GFP*, *35S::PagWUS2-GFP*, or *35S::CBL1-RFP* (kindly provided by Sha Li, Shandong Agricultural University) vector was transformed into fully expanded leaves of *N. benthamiana* via *Agrobacterium tumefaciens* GV3101^[56]^. The *35S::PagCLV3-1-GFP* and *35S::PagCLV3-2-GFP* vectors were co-infiltrated with the plasma membrane marker *35S::CBL1-RFP*. After incubation in the dark for 48 h, fluorescence was viewed and imaged under a Zeiss LSM 880 NLO confocal microscope (Carl Zeiss, Jena, Germany). The GFP signal was detected at 488 nm, and the DAPI signal at 405 nm^[57]^.

### GUS staining

Shoot tips of *pPagCLV3-1::GUS* saplings were fixed in 90% [v/v] acetone on ice for 20 min. After washing twice with a buffer containing 50 mM NaPO_4_ (pH 7.2), 0.5 mM K_3_Fe (CN)_6_, and 0.5 mM K_4_Fe (CN)_6_, the tissue was immersed in β-glucuronidase (GUS) staining buffer containing 2 mM X-Gluc and vacuum-infiltrated for 10 min. After incubation at 37 °C for 12 h, the stained material was washed with 70% ethanol solution for 12 h, then embedded in low-melting-point agarose and sectioned. The sections were viewed and imaged under an Olympus BX-51 microscope.

### Confocal microscopy

Shoot tips were fixed in 2.5% (v/v) paraformaldehyde on ice and vacuum-infiltrated for 30 min. After fixing at 4 °C overnight, the tissue was successively washed with 10%, 20%, and 30% sucrose solutions (dissolved in 1% [v/v] paraformaldehyde). The tissues were then embedded in low-melting-point agarose, sectioned, and viewed and imaged under a Zeiss LSM 880 NLO confocal microscope (Carl Zeiss). The GFP signal was detected with excitation at 488 nm and emission at 505–550 nm. The cell outline was stained with Fluorescent Brightener, and its signal was detected with excitation at 405 nm, and emission at 425–475 nm^[58]^.

## Results

### *PagWUS1/2* and *PagCLV3-1/2* exhibit similar functions with their respective orthologs *AtWUS* and *AtCLV3*

To identify the putative orthologs of Arabidopsis *WUS* and *CLV3* in poplar, we used the protein sequences of AtWUS and AtCLV3 as queries in Blast searches against the genome of Poplar 84K (*Populus alba* × *Populus tremula* var. *glandulosa*) (Supplementary Table S2). Consistent with a previous report, two sequences demonstrated the highest conservation with AtWUS, and were thus annotated as PagWUS1 and PagWUS2 (Supplementary Fig. S2)^[59]^. Two CLAVATA3/Embryo Surrounding Region-related (CLE) family proteins containing putative N-terminal signal peptides showed the highest similarity to AtCLV3, and were annotated as PagCLV3-1 and PagCLV3-2 (Supplementary Fig. S3, Supplementary Table S3)^[60−62]^.

We expressed *PagWUS1* under the control of the *AtWUS* promoter in the Arabidopsis *wus-1* mutant^[63]^. The shoot development phenotype of *wus-1* seedlings was largely rescued (Supplementary Fig. S4a, S4b), and the defects in the floral organs of *wus-1* were also substantially complemented (Supplementary Fig. S4c). Introduction of the *pAtCLV3::PagCLV3-2* vector into the *clv3-2* mutant partially rescued its defective seedling, silique, and flower development (Supplementary Fig. S5). The above results indicate that *PagWUS1* and *PagCLV3-2* have similar functions to *AtWUS* and *AtCLV3*, respectively.

### *PagWUS1/2* and *PagCLV3-1/2* are specifically expressed in the shoot meristem

**Figure 1:**
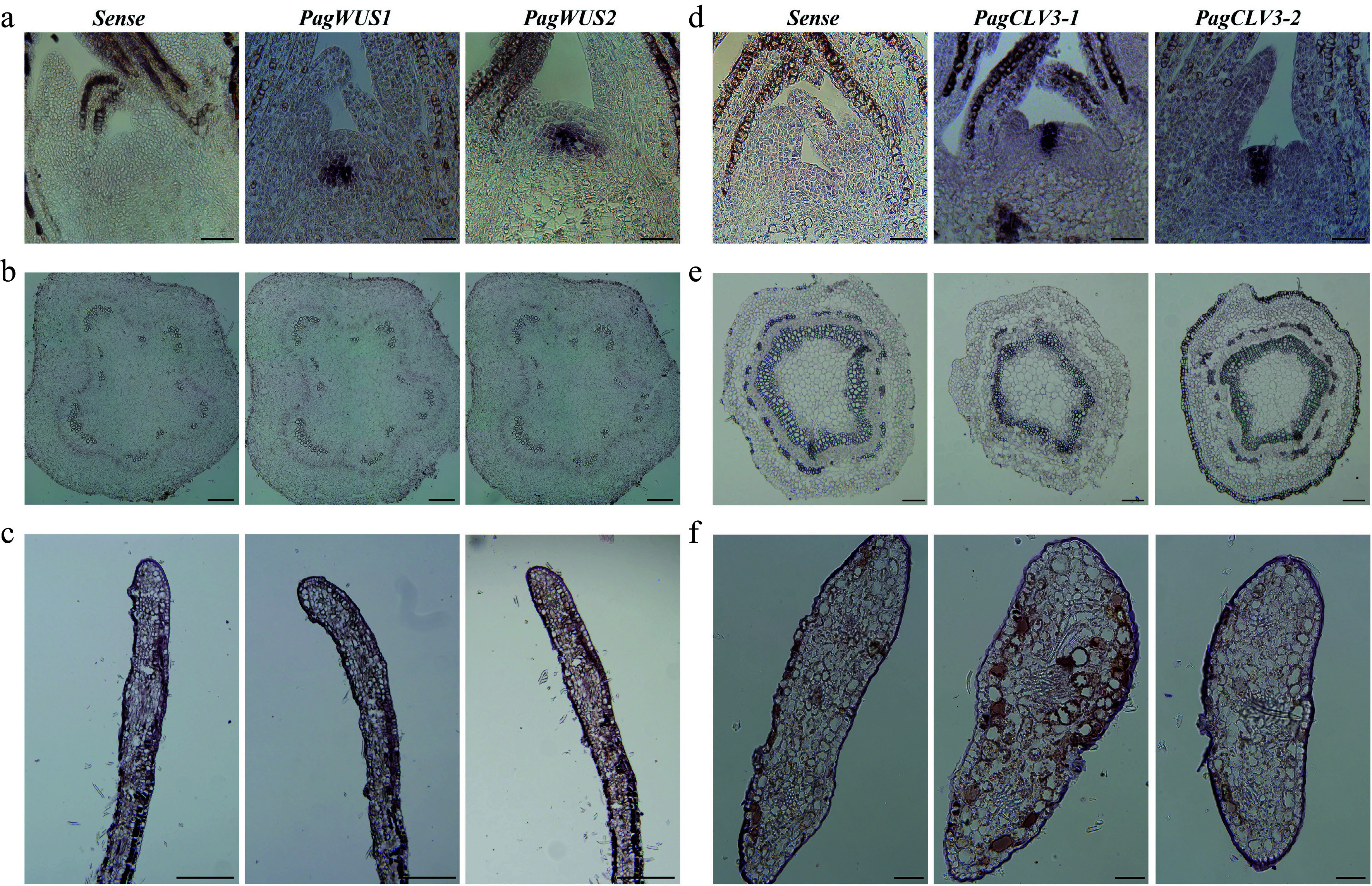
Expression patterns of *PagWUS1/2* and *PagCLV3-1/2* as detected by *in situ* hybridization. Signals of *PagWUS1* and *PagWUS2* transcripts were (a) present in the organizing center (OC) of shoot meristem, but (b) absent from the cross section of third internode, and (c) longitudinal section of young leaf. Signals of *PagCLV3-1* and *PagCLV3-2* transcripts were (d) localized in the central zone (CZ) of shoot meristem, but (e) absent from the cross section of fifth internode, and (f) cross section of young leaf. Sense probe controls are shown in the left column. Bar = 100 μm.

To examine the distribution of *PagWUS1/2* and *PagCLV3-1/2* transcripts, *in situ* hybridization was performed using cross sections and longitudinal sections of the meristem, internodes, and leaves. Transcripts of both *PagWUS1* and *PagWUS2* were present in the OC of the shoot meristem, but not in the cross section of the third internode and the longitudinal section of the young leaf (Fig. 1a–c). The *PagCLV3-1* and *PagCLV3-2* transcript signals were specifically localized in the CZ of the shoot meristem, but were not visible in the fifth internode or the cross section of the young leaf (Fig. 1d–f).

In the Arabidopsis shoot meristem, *AtCLV3* is expressed in the center of the outermost three cell layers, while *AtWUS* is expressed in the three-layered OC beneath^[6,10]^. However, the *PagCLV3-1/2* signals were present in six cell layers, and *PagWUS1/2* signals were present in four to five cell layers (Supplementary Fig. S6a–S6d). Cells expressing *AtCLV3* and *AtWUS,* which constitute the stem cell niche in Arabidopsis, accounted for 10.1% of the total cell number of the shoot meristem (Supplementary Fig. S6e), whereas this proportion was much higher (20.9%) in poplar. Moreover, the ratios of height, width, and area of the *PagCLV3-1/PagWUS*-expressing domain to those of the poplar meristem were significantly higher than those of the *AtCLV3/AtWUS*-expressing domain to the Arabidopsis meristem (Supplementary Fig. S6f, S6g). Estimates based on analyses of the 2D sections indicate that, during vegetative growth under normal conditions, the stem cell niche accounts for a larger proportion of the meristem in poplar than in Arabidopsis.

We then tried to visualize the expression patterns of *PagWUS* and *PagCLV3* using reporter lines. The 2,408-bp or 6,186-bp sequences immediately upstream of the ATG start codon of *PagWUS2* were used as promoters to generate the *PagWUS2::GFP*_*3*_ and *PagWUS2::GFP*_*3*_-L lines, respectively. The results show that GFP signals were distributed beyond the OC into the PZ or leaf primordium (Supplementary Fig. S7a, S7b). The 4,707-bp upstream sequence of *PagCLV3-1* gave rise to expression signals in the OC (Supplementary Fig. S7c). After adding the 1,791-bp sequence downstream of the translational stop codon, GFP signals were still mainly distributed in the OC (Supplementary Fig. S7d). These results suggest that the tested regulatory fragments of *PagWUS2* and *PagCLV3-1* may be insufficient. Additional approaches are required to capture the authentic promoter regions.

To determine the subcellular localization of PagWUS1 and PagWUS2, *35S::PagWUS1-GFP* and *35S::PagWUS2-GFP* vectors were separately transformed into tobacco leaf epidermal cells. Consistent with their proposed roles as transcription factors, the PagWUS1-GFP and PagWUS2-GFP signals overlapped with those of DAPI, confirming their localization in the nucleus (Supplementary Fig. S8a). We next co-infiltrated the *35S::PagCLV3-1-GFP* and *35S::PagCLV3-2-GFP* vectors with the plasma membrane marker *35S::CBL1-RFP*, respectively^[56]^. Both the GFP and RFP signals were localized along the cellular outline (Supplementary Fig. S8b). To determine whether PagCLV3-1/2 were localized in the plasma membrane or exported into the extracellular space, tobacco leaves were treated with a sucrose solution to induce plasmolysis. The PagCLV3-1-GFP and PagCLV3-2-GFP signals were partially separated from CBL1-RFP and localized in the apoplast, indicating that PagCLV3-1 and PagCLV3-2 are secreted proteins.

### *PagWUS1/2* and *PagCLV3-1/2* regulate the maintenance of the shoot meristem by forming a feedback loop

**Figure 2:**
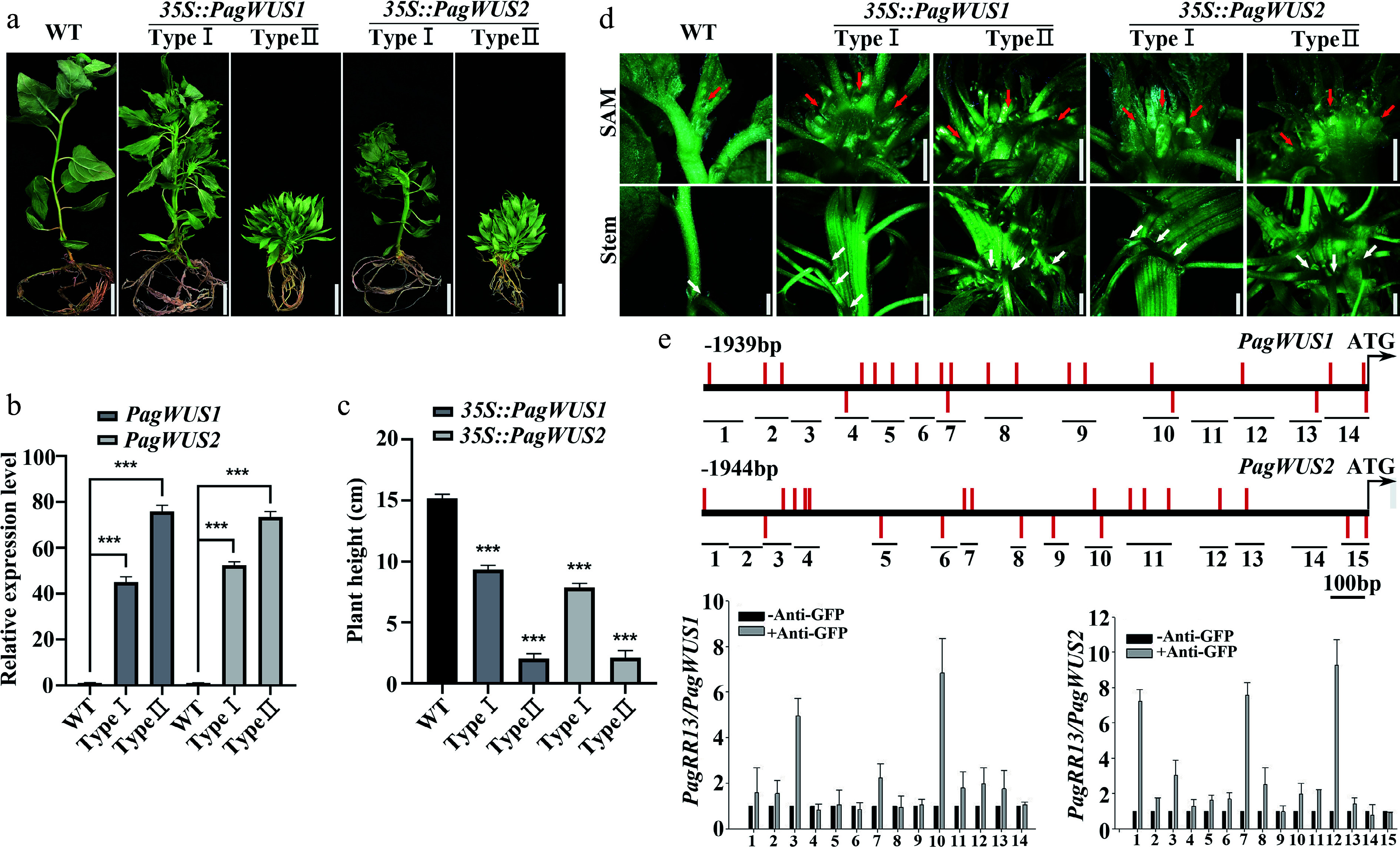
*PagWUS1/2* positively regulate shoot meristem activity. (a) Phenotype of *35S::PagWUS1* and *35S::PagWUS2* seedlings. Bar = 2 cm. (b) Correlation between relative transcript levels of *PagWUS1/2* and phenotype severity of overexpressing lines. (c) Plant height of *35S::PagWUS1* and *35S::PagWUS2* saplings. (d) Fasciated shoot meristem and stem with disordered phyllotaxis in *35S::PagWUS1* and *35S::PagWUS2* lines. Red arrows indicate fasciated shoot meristem. White arrows denote disordered phyllotaxis. Bar = 2 mm. (e) ChIP-qPCR results showing association between PagRR13 and *PagWUS1/2* promoter fragments. Scheme of promoter regions of *PagWUS1* and *PagWUS2* is shown above. Black short lines with numbers indicate positions of corresponding fragments used for ChIP-qPCR. Red bars indicate type-B RR binding elements GAT(T/C). Data are mean ± sd of three independent biological repeats. *** Indicates significant difference at *p* < 0.001 (two-tailed Student’s t-tests).

To study the biological functions of *PagWUS1* and *PagWUS2*, we first overexpressed these two genes under the control of the cauliflower mosaic virus 35S promoter. There were two phenotypes among the *35S::PagWUS1/2* lines (Fig. 2a, b). In total, we obtained four Type I and three Type II *35S::PagWUS1* lines, and four Type I and four Type II *35S::PagWUS2* lines. Different lines from the same type exhibited consistent and stable phenotypes. We selected two lines with different phenotypic types of *35S::PagWUS1* and *35S::PagWUS2*, respectively, for further analysis. Compared with the wild type, the Type I lines with relatively lower *PagWUS1/2* transcript levels grew slowly with reduced plant height (Fig. 2a, c). The shoot meristem was fasciated, which generated a fasciated stem with disordered phyllotaxis, indicating the production of supernumerary meristematic cells (Fig. 2d). The Type II lines demonstrated stronger phenotypes in terms of growth retardation and stem fasciation, consistent with the relatively higher *PagWUS1/2* transcript levels.

Neither the *CRISPR-PagWUS1* line nor the *CRISPR-PagWUS2* line showed any visible phenotype, possibly because of functional redundancy. We were unable to obtain *CRISPR-PagWUS1-2* saplings, suggesting that disruption of both genes may compromise shoot regeneration (Supplementary Fig. S1).

Additionally, ChIP-qPCR analysis revealed that PagRR13 (a homolog of the AtARR1 transcription factor) associated with the promoter regions of both *PagWUS1* and *PagWUS2* (Fig. 2e). The results of the dual-luciferase assays showed that PagRR13 activated the expression of *PagWUS1/2*, indicative of direct regulation between cytokinin signaling and *PagWUS1/2* transcription (Supplementary Fig. S9).

**Figure 3:**
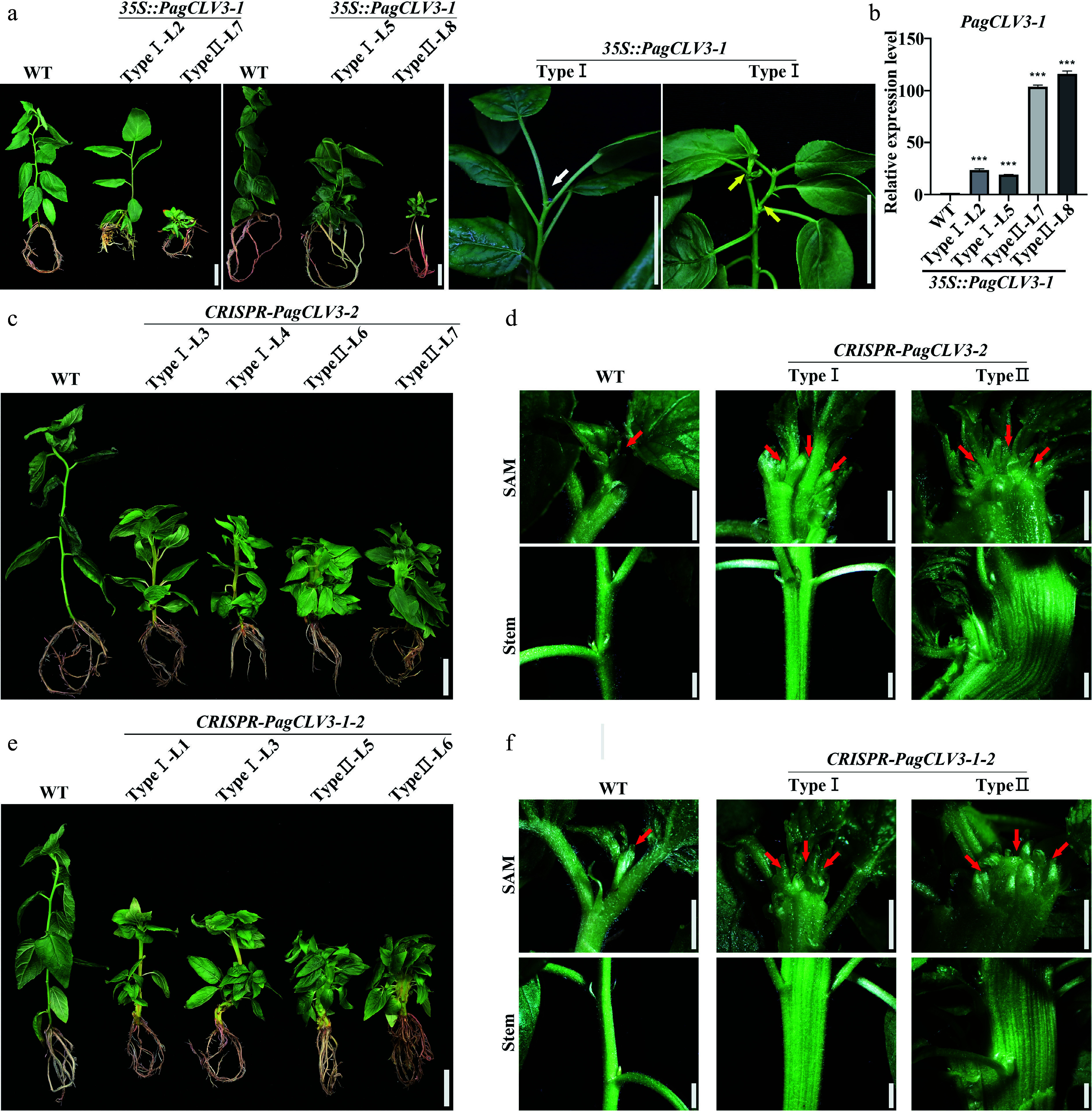
*PagCLV3* negatively regulates shoot meristem maintenance. (a) Phenotype of *35S::PagCLV3-1* seedings. White arrow denotes position of vanished shoot meristem. Yellow arrows indicate axial bud outgrowths. (b) Correlation between relative transcript levels of *PagCLV3-1* in *35S::PagCLV3-1* lines and phenotype severity. Data are mean ± sd of three independent biological repeats. (c) Phenotype of *CRISPR-PagCLV3-2* seedlings. (d) Fasciation phenotypes of saplings in (c). (e) Phenotype of *CRISPR-PagCLV3-1-2* (f) Fasciation phenotypes of saplings in (e). In (d) and (f), red arrows indicate shoot meristem. For (a), bar = 2 cm. For (c) and (e), bar = 2 cm. For (d) and (f), bar = 2 mm. Data are mean ± sd of three independent biological repeats. *** indicates significant difference at *p* < 0.001 (two-tailed Student’s t-tests).

We further explored the functions of *PagCLV3-1/2* through overexpression and knock-out (CRISPR/Cas9) strategies. The *35S::PagCLV3-1/2*-overexpressing lines exhibited two types of phenotypes (we obtained five Type I and three Type II *35S::PagCLV3-1* lines, and six Type I and three Type II *35S::PagCLV3-2* lines) (Fig. 3a, b; Supplementary Fig. S10). For each type, two independent lines were selected for phenotypic analysis. The saplings with relatively lower *PagCLV3-1/2* transcript levels grew in a 'stop-go' manner (Type I). After a period of growth, the shoot meristem vanished, and the meristematic cells were consumed (Fig. 3a; Supplementary Figs S11 and S12). About 10 d later, the shoot meristem was reformed. During this process, a number of axial buds grew out and formed branches, indicating compromised apical dominance resulting from the cessation of meristem activity. In saplings with relatively higher *PagCLV3-1/2* transcript levels (Type II), shoot meristem activity ceased at early stages, resulting in a disorganized bunch of leaves and no stem elongation (Fig. 3a, b). Therefore, overexpression of *PagCLV3-1/2* repressed shoot meristem activity.

In contrast, the *CRISPR-PagCLV3-2* saplings (eight Type I and three Type II lines) generated a fasciated shoot meristem, leading to a fasciated stem and random phyllotaxis (Fig. 3c, d; Supplementary Figs S1 and S13). To confirm that the fasciation and phyllotaxis defects in *CRISPR-PagCLV3-2* mutants resulted from the loss of *PagCLV3-2*, we applied a synthetic PagCLV3-2 peptide (0.1, 1, and 10 μM) to mutate shoot apices *in vitro*. This largely rescued the fasciation and phyllotaxis phenotypes (Supplementary Fig. S14). Mutation of both *PagCLV3-1* and *PagCLV3-2* in the *CRISPR-PagCLV3-1-2* lines (nine Type I and four Type II lines) resulted in more severe phenotypic defects than those of single mutants (Fig. 3e, f). Thus, similar to overexpression of *PagWUS1/2*, defects in *PagCLV3-1/2* led to supernumerary meristematic cells.

**Figure 4:**
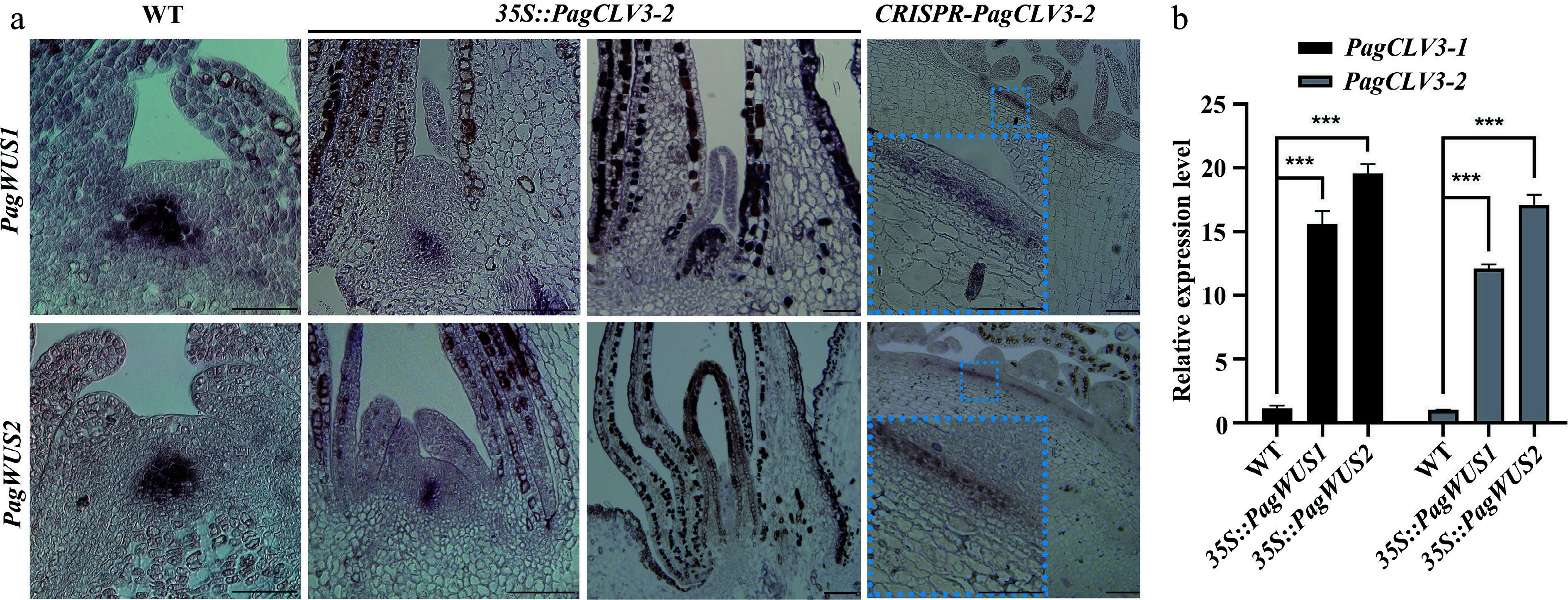
Negative feedback regulation between transcription of *PagWUS* and *PagCLV3*. (a) *In situ* hybridization of *PagWUS1* and *PagWUS2* transcripts in shoot meristem of wild-type, *35S::PagCLV3-2,* and *CRISPR-PagCLV3-2* saplings. Lower left insets in the right column are magnified images of blue dotted rectangles. Bar = 100 μm. (b) qRT-PCR analyses showing increased *PagCLV3-1/2* transcript levels in shoot tips of *35S::PagWUS1* and *35S::PagWUS2* saplings. Data are mean ± sd of three independent biological repeats. *** Indicates significant difference at *p *< 0.001 (two-tailed Student’s t-tests).

To further determine the regulatory relationship between *PagWUS1/2* and *PagCLV3-1/2* transcription, we examined the transcript signals of *PagWUS1/2* in the *35S::PagCLV3-2* shoot meristem by *in situ* hybridization. The results show that *PagWUS1/2* transcripts were present at considerably reduced levels in the active meristem and undetectable in the inactive meristem (Fig. 4a). In the fasciated *CRISPR-PagCLV3-2* shoot meristem, *PagWUS1/2* were ectopically expressed in a band-like region beneath the outermost two cell layers (Fig. 4a). These results show that PagCLV3-1/2 negatively regulate the expression of *PagWUS1/2*. As determined by qRT-PCR analyses, the transcript levels of *PagCLV3-1/2* were significantly increased in the shoot tips of both *35S::PagWUS1* and *35S::PagWUS2* saplings, indicating that PagWUS1/2 activate the transcription of *PagCLV3-1/2* (Fig. 4b). Together, these results show that *PagWUS1/2* and *PagCLV3-1/2* maintain the shoot meristem by forming a feedback loop.

### Involvement of the *PagWUS-PagCLV3* module in shoot regeneration

**Figure 5:**
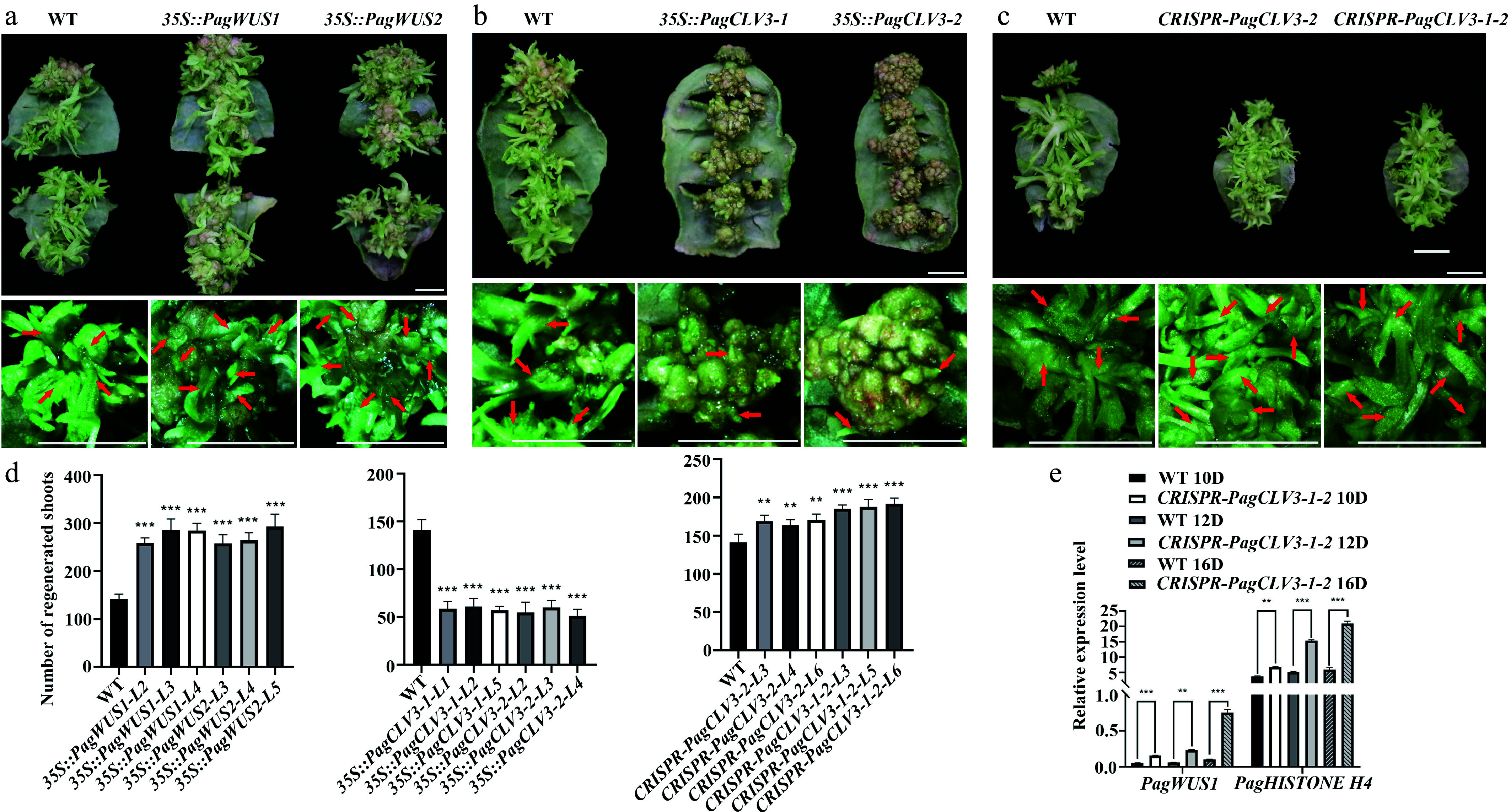
Participation of *PagWUS-PagCLV3* module in shoot regeneration. Shoot regeneration was promoted in (a) *35S::PagWUS*, repressed in (b) *35S::PagCLV3*, and enhanced in the (c) *CRISPR-PagCLV3* explants. Bars = 5 mm. For (a)–(c), upper panels show shoots regenerated from leaf explants. Lower panels show formation of regenerated shoots. Red arrows indicate position of regenerated shoots. (d) Numbers of regenerated shoots per leaf explant. For *35S::PagWUS1*, *n* = 25; *35S::PagWUS2*, *n* = 25; *35S::PagCLV3-1*, *n* = 25; *35S::PagCLV3-2*, *n* = 25; *CRISPR-PagCLV3-2*, *n* = 25; *CRISPR-PagCLV3-1-2*, *n* = 25. Error bars show mean ± sd. (e) qRT-PCR analyses showing increased *PagWUS1* and *Histone H4* transcript levels in *CRISPR-PagCLV3-1-2* explants, as compared with wild type. D indicates days of culture. Data are mean ± sd of three independent biological repeats. Asterisks indicate significant differences: ** 0.001 < *p* < 0.01 and *** *p *< 0.001 (two-tailed Student’s t-tests).

Next, we examined whether *PagWUS1/2* and *PagCLV3-1/2* participate in shoot regeneration. When wild-type leaves were used as explants, an average of 141 shoots were produced from one leaf after 30 d of incubation (Fig. 5a, d). In the *35S::PagWUS1* and *35S::PagWUS2* lines, this number was increased to 285 and 293, respectively. By contrast, overexpression of *PagCLV3-1* or *PagCLV3-2* significantly reduced shoot regeneration (Fig. 5b, d).

Unexpectedly, the *CRISPR-PagCLV3-2* and *CRISPR-PagCLV3-1-2* lines also showed enhanced shoot regeneration capacity (Fig. 5c, d). By contrast, the Arabidopsis *clv3* mutant did not show a similar phenotype (Supplementary Fig. S15). Previous studies revealed that CLV3 negatively regulates *WUS* expression and restricts cell division^[64]^. We thus postulate that during the reestablishment of the shoot meristem, the defective *PagCLV3-1/2* released the constraints on *PagWUS1/2* expression and cell proliferation, and thus promoted shoot meristem formation. To test this hypothesis, we examined the transcript levels of *PagWUS1* and *Histone H4* (a marker for cell division) by qRT-PCR (Fig. 5e; Supplementary Fig. S16). The results show that, compared with the wild type, the transcript levels of *PagWUS1* and *H4* were significantly increased at day 10 of culture on SIM (SIM10), SIM12, and SIM16.

### *PagWUS-PagCLV3* module controls shoot meristem cessation

**Figure 6:**
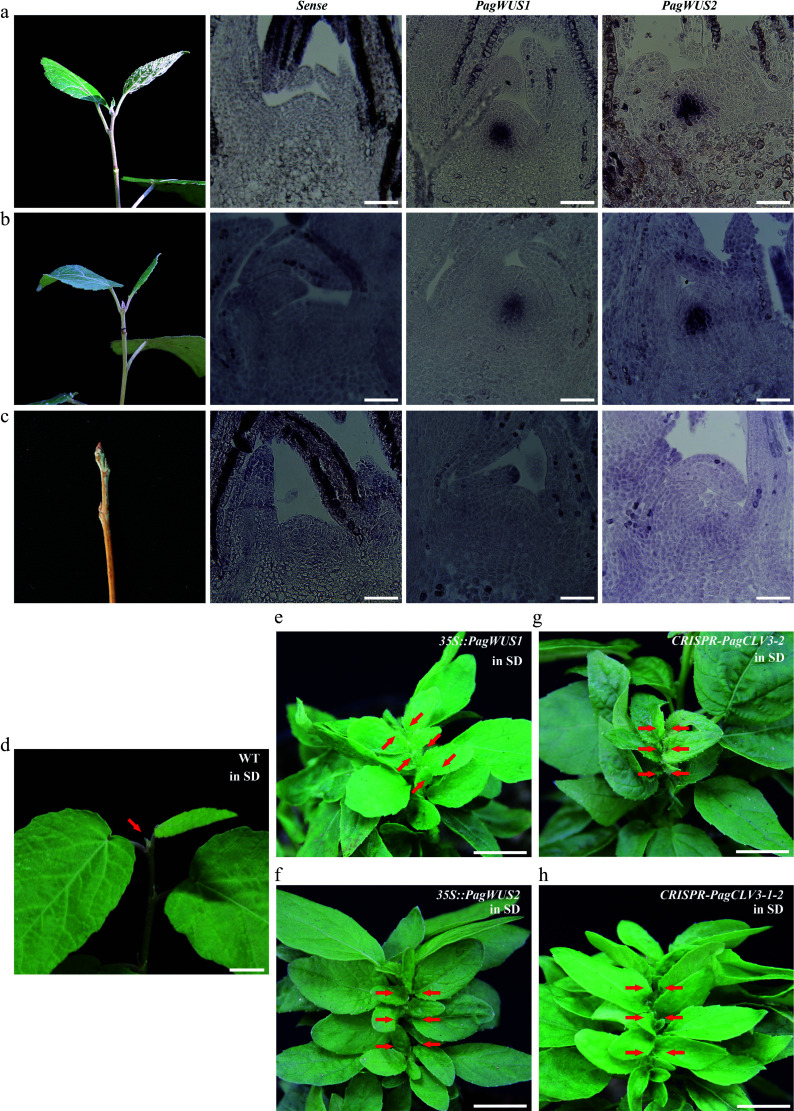
The *PagWUS-PagCLV3* module regulates the cessation of shoot meristem activity. *In situ* hybridization analyses showing abundance of *PagWUS1* and *PagWUS2* transcripts: (a) abundant during growth season, (b) reduced at the onset of cessation, and (c) absent after bud scale maturation. Bar = 100 μm. After short day treatment for 4 weeks, shoot meristem became inactive in (d) wild-type, but remained active in (e) *35S::PagWUS1*, (f) *35S::PagWUS2*, (g) *CRISPR-PagCLV3-2*, and (h) *CRISPR-PagCLV3-1-2* saplings. Red arrows indicate shoot meristem. Bar = 1 cm.

Next, we investigated whether *PagWUS1/2* expression is responsive to seasonal changes. For this purpose, *in situ* hybridization analyses were performed on shoot meristems collected in different seasons. The results show that both *PagWUS1* and *PagWUS2* were actively expressed during the growth season (Fig. 6a–c). However, the transcript signals were obviously weaker at the onset of growth cessation and bud set, and completely absent after bud scale maturation. Thus, the transcript levels of *PagWUS1* and *PagWUS2* were correlated with the shift in meristem activity toward dormancy.

We wondered whether the *PagWUS-PagCLV3* module is implicated in regulating the cessation of shoot meristem activity. To address this question, we first determined whether growth cessation of the shoot is controlled by day length or temperature. Consistent with previous studies, we found that short-day (8-h light/16-h dark photoperiod) but not low temperature (4 °C) conditions for 7 weeks led to growth cessation in wild-type saplings (Supplementary Fig. S17)^[65]^. Next, we tested whether overexpression of *PagWUS1/2* or disruption of *PagCLV3-1-2* affected the cessation of meristem activity. Saplings of the *35S::PagWUS1/2*, *CRISPR-PagCLV3-2,* and *CRISPR-PagCLV3-1-2* lines were cultured under short-day conditions. After 4 weeks, meristem activity had ceased in the wild-type but persisted in all the transgenic lines, which continually formed new leaves (Fig. 6d–h; Supplementary Fig. S18). These results indicate that *PagWUS1/2* positively regulates meristem activity, whereas *PagCLV3-1/2* has the opposite role.

### *PagWUS-PagCLV3* module mediates proper pattern of secondary growth

Because mutation of *PagCLV3-1/2* or overexpression of *PagWUS1/2* gave rise to a fasciated stem, we wondered whether secondary growth was affected in these lines. To address this question, we compared the histological structure of the stem between the wild-type and *CRISPR-PagCLV3-1-2* and *35S::PagWUS1* lines. In the cross sections of the wild-type stem, vascular bundles were evenly distributed in the fourth internode, while the secondary phloem and xylem were radially thickened in the tenth internode (Supplementary Fig. S19a, S19b). In the *CRISPR-PagCLV3-1-2* stem, it was difficult to distinguish two adjacent internodes because of the disrupted phyllotaxis. Therefore, we observed cross sections of the upper (5 mm from the shoot tip) and lower (5 cm from the shoot tip) parts of the stem. In the upper part, vascular bundles were evenly distributed in the fasciated stem (Supplementary Fig. S19c). However, in contrast to the wild-type’s stem, that of the *CRISPR-PagCLV3-1-2* mutant showed uneven thickening in the lower part, i.e., the secondary phloem and xylem were clearly formed at one end, but the other end was still in the primary growth stage (Supplementary Fig. S19d). The *35S::PagWUS1* stem demonstrated a similar histological structure to that of *CRISPR-PagCLV3-1-2* (Supplementary Fig. S19e, S19f). These results show that the *PagWUS-PagCLV3* module mediates the proper pattern of secondary growth by maintaining the shoot meristem.

## Discussion

More than two decades ago, the WUS-CLV3 module was established as the pivotal circuit regulating the balance between stem cell homeostasis and differentiation of the shoot meristem^[6,10]^. Since then, *WUS* and *CLV3* orthologs have been characterized and analyzed in a number of herbaceous species. Their biological roles have been found to be largely conserved in dicots, including Arabidopsis, *Brassica rapa*, tomato (Solanum lycopersicum), *Medicago truncatula*, *Antirrhinum majus*, and petunia (*Petunia hybrida*)^[9,66−71]^. However, the regulatory roles of the WUS-CLV3 module in perennial woody plants remain unknown.

In this study, we investigated the functions of the *PagWUS-PagCLV3* module. The results demonstrate conserved as well as deviated functions compared with those of their orthologs in herbaceous species. *PagWUS* and *PagCLV3* maintain the shoot meristem by forming a feedback loop, similar to the case in herbaceous species^[72]^.

Different from its herbaceous orthologs, the *PagWUS-PagCLV3* module is consistent with the developmental characteristics of perennial trees by regulating bud dormancy and secondary growth. In perennial plants, bud dormancy is a strategy to cope with seasonal changes^[39]^. The induction and signal transduction mechanisms related to dormancy have been studied in detail^[48]^. Despite these studies, how shoot meristem activity is regulated during the induction of dormancy remains elusive. We found that the transcript levels of *PagWUS1/2* were correlated with meristem activity in poplar. Their transcript signals were abundant during the growth season, reduced at the onset of growth cessation, and completely absent after bud scale maturation (Fig. 6). Furthermore, overexpression of *PagWUS1/2* or disruption of *PagCLV3-1/2* delayed bud dormancy. These findings suggest that the *PagWUS-PagCLV3* module acts as the mediator between dormancy regulators and shoot meristem activity.

Moreover, disrupting the *PagWUS-PagCLV3* module gave rise to a fasciated stem in which the progress of secondary growth was uneven (Supplementary Fig. S19). We speculate that this phenotype was caused by the disruption of the radial distribution of hormones such as cytokinin and auxin, which are essential for cambium activity^[73]^. Whether the distribution patterns of these hormones and their signaling transduction are directly regulated by *PagWUS* or *PagCLV3* is unknown. However, our results show that the *PagWUS-PagCLV3* module influences the proper pattern of secondary growth in poplar through maintaining the appropriate structure and function of the shoot meristem.

Previous studies reported that overexpression of *WUS* facilitated the formation of somatic embryo and shoot structure^[74−76]^. In contrast, whether and how *CLV3* is involved in shoot regeneration remained unknown. Consistently, our results show that shoot regeneration in poplar was significantly enhanced in the *35S::PagWUS* lines. However, whereas the Arabidopsis *clv3* single mutant did not show any changes in shoot regeneration phenotype, the *CRISPR-PagCLV3* lines showed a clearly enhanced regeneration capacity. Because *PagCLV3* and *AtCLV3* have similar molecular functions (Supplementary Figs S5, S15), we speculate that the regulatory network of shoot regeneration differs between poplar and Arabidopsis. The results imply that silencing of *CLV3* may be a strategy for establishing a high-efficiency shoot regeneration system in trees.

To our knowledge, this is the first report on the function of the WUS-CLV3 module in a perennial woody plant. The results reveal that the *PagWUS-PagCLV3* module plays a basic role in regulating homeostasis of the shoot meristem in a manner conserved among dicots, and mediates the proper pattern of secondary growth and bud dormancy consistent with the typical characteristics of perennial trees. Our findings provide new information about the function and regulatory mechanisms of the shoot meristem in perennial woody species.

## Conclusions

We investigated the functions of the poplar genes *PagWUS* and *PagCLV3*, homologs of Arabidopsis *WUS* and *CLV3*, respectively. Similar to their orthologs in dicotyledonous herbs, *PagWUS* and *PagCLV3* form a feedback loop that regulates shoot meristem maintenance. Overexpression of *PagWUS* promoted shoot regeneration. Compared with herbaceous species, poplar has a much larger stem cell niche. Disruption of *PagCLV3* enhanced the shoot regeneration capacity. Our results show that the *PagWUS-PagCLV3* module controls the cessation of shoot meristem activity and mediates the proper pattern of secondary growth. These findings provide insights into the mechanisms of shoot meristem regulation in trees.

## SUPPLEMENTARY DATA

Supplementary data to this article can be found online.

## Data Availability

All the data supporting the findings of this study are available in the paper and supplementary data.
