# Supplementary material for: The PagWUS-PagCLV3 module regulates shoot meristem maintenance and activity in poplar
Source: For Res (Fayettev). 2026 Mar 26;6:e007. doi: 10.48130/forres-0026-0007 (PMC13191361; doi:10.48130/forres-0026-0007)
Supplement: Supplementary file 1 — Supplementary data to this article can be found online. [file FR-2026-6-007-S1.zip › 10.48130_forres-0026-0007-Suppl-FigureS1.pdf]

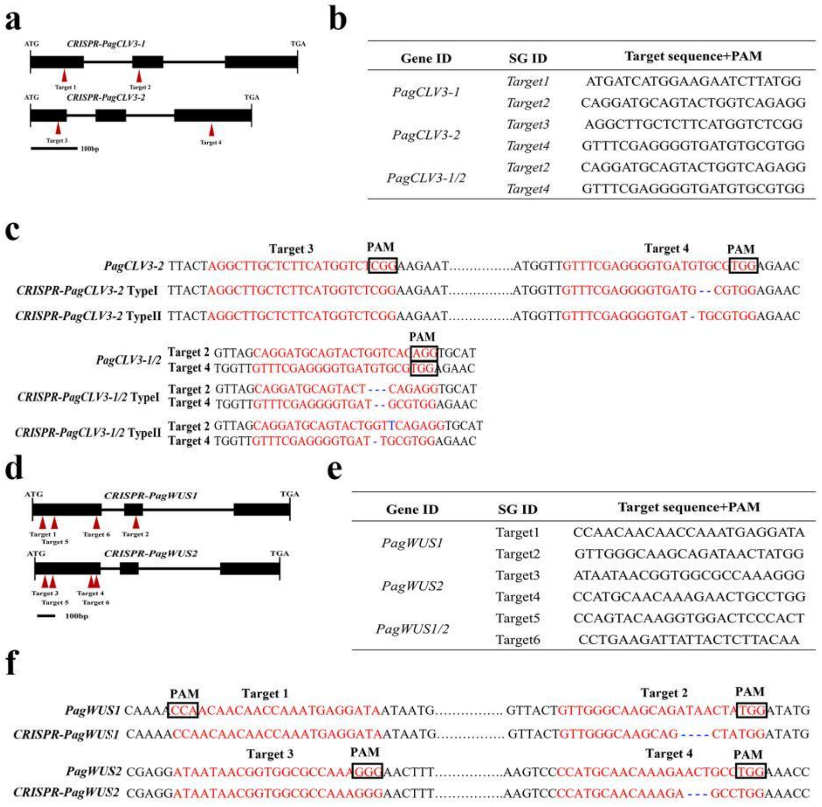

Supplementary Fig. S1

Targets of *CRISPR-PagCLV3* and *CRISPR-PagWUS* lines. (a) and (d), schematic diagram shows the positions of *CRISPR-PagCLV3* (a) and *CRISPR-PagWUS* (d) targets. (b) and (e), sequences of target positions of *CRISPR-PagCLV3* (b) and *CRISPR-PagWUS* (e). (c) and (f), sequence changes surrounding the targets positions of *CRISPR-PagCLV3* (c) and *CRISPR-PagWUS* (f). Red letters represent sgRNA sequences. Black boxes denote protospacer-adjacent motif (PAM) sequences. A 2-bp and a 1-bp deletions were identified at target site 4 in Type I and Type II *CRISPR-PagCLV3-2* lines, respectively. A 3-bp and a 2-bp deletions were identified at target site 2 and target site 4 in Type I *CRISPR-PagCLV3-1-2* line, respectively. A 1-bp insertion and a 1-bp deletion were identified at target site 2 and target site 4 in Type II *CRISPR-PagCLV3-1-2* line, respectively. A 4-bp deletions were identified at target site 2 in *CRISPR-PagWUS1* line. A 3-bp deletions were identified at target site 4 in *CRISPR-PagWUS1* line. Blue dashed lines represent base deletion. Blue letter indicates inserted base.
