# Supplementary material for: The PagWUS-PagCLV3 module regulates shoot meristem maintenance and activity in poplar
Source: For Res (Fayettev). 2026 Mar 26;6:e007. doi: 10.48130/forres-0026-0007 (PMC13191361; doi:10.48130/forres-0026-0007)
Supplement: Supplementary file 1 — Supplementary data to this article can be found online. [file FR-2026-6-007-S1.zip › 10.48130_forres-0026-0007-Suppl-FigureS10.pdf]

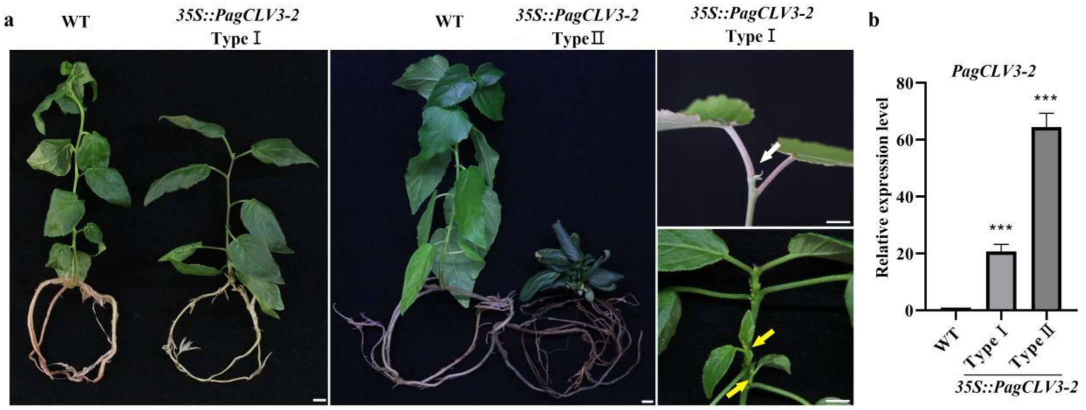

Supplementary Fig. S10

PagCLV3-2 negatively regulated shoot meristem homeostasis. (a) Shoot meristem activity was suppressed in Type I *35S::PagCLV3-2* saplings (left panel). Shoot meristem of Type II *35S::PagCLV3-2* saplings ceased at early stages and gave rise to a disorganized bunch of leaves without stem elongation (middle panel). In the right panels, White arrow denotes the position of vanished shoot meristem. Yellow arrows indicate axially bud outgrowth. (b) Relative transcript levels of *PagCLV3-2* in *35S::PagCLV3-2* lines correlated with the extent of phenotype. Bar = 1 cm. Data are mean ± s.d. of three independent biological repeats. ***P < 0.001 are determined by two-tailed Student's t-tests.
