# Supplementary material for: The PagWUS-PagCLV3 module regulates shoot meristem maintenance and activity in poplar
Source: For Res (Fayettev). 2026 Mar 26;6:e007. doi: 10.48130/forres-0026-0007 (PMC13191361; doi:10.48130/forres-0026-0007)
Supplement: Supplementary file 1 — Supplementary data to this article can be found online. [file FR-2026-6-007-S1.zip › 10.48130_forres-0026-0007-Suppl-FigureS11.pdf]

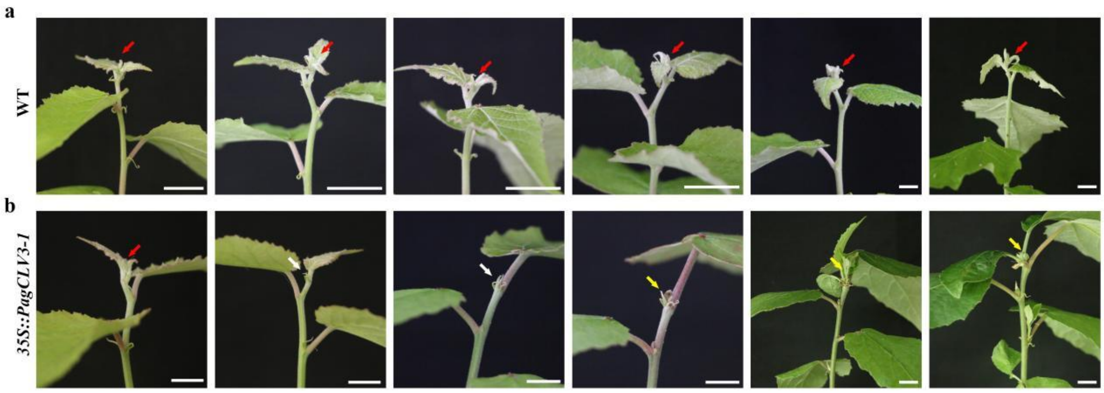

Supplementary Fig. S11

The “stop-and-go” growth manner of Type I *35S::PagCLV3-1* saplings. (a) Wild type saplings maintained normal shoot meristem. (b) Shoot meristem of Type I *35S::PagCLV3-1* saplings ceased after a period of growth, and was reformed about 10 days later.. Successive growth stages with 10 days-interval are shown from left to right. Red arrows indicate shoot meristem. White arrow denotes the position of vanished shoot meristem. Yellow arrows indicate regenerated shoot meristem. Bar = 2 cm.
