# Supplementary material for: The PagWUS-PagCLV3 module regulates shoot meristem maintenance and activity in poplar
Source: For Res (Fayettev). 2026 Mar 26;6:e007. doi: 10.48130/forres-0026-0007 (PMC13191361; doi:10.48130/forres-0026-0007)
Supplement: Supplementary file 1 — Supplementary data to this article can be found online. [file FR-2026-6-007-S1.zip › 10.48130_forres-0026-0007-Suppl-FigureS12.pdf]

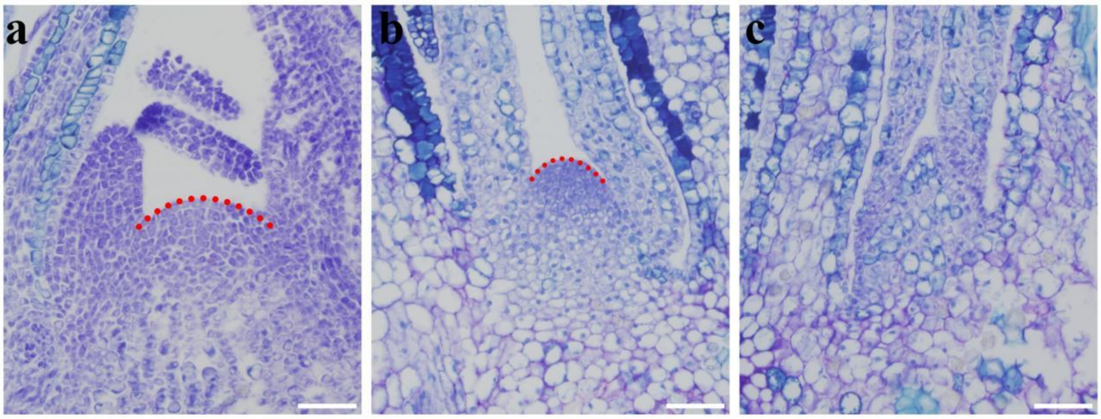

Supplementary Fig. S12

The process of shoot meristem consumption in Type I *35S::PagCLV3-1* saplings. Histological analysis of active (a), compromised (b) and consumed (c) shoot meristems in Type I *35S::PagCLV3-1* saplings. Outline of meristem surface is marked by red dotted line. Bars = 100 μm.
