# Supplementary material for: The PagWUS-PagCLV3 module regulates shoot meristem maintenance and activity in poplar
Source: For Res (Fayettev). 2026 Mar 26;6:e007. doi: 10.48130/forres-0026-0007 (PMC13191361; doi:10.48130/forres-0026-0007)
Supplement: Supplementary file 1 — Supplementary data to this article can be found online. [file FR-2026-6-007-S1.zip › 10.48130_forres-0026-0007-Suppl-FigureS13.pdf]

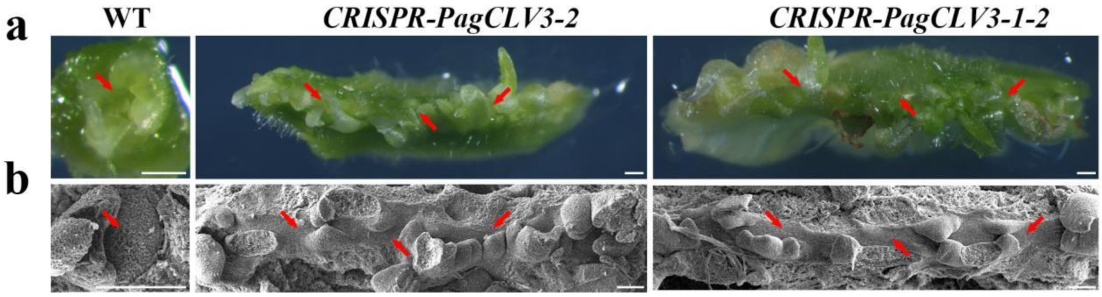

Supplementary Fig. S13

Morphology of fasciated shoot meristem in *CRISPR-PagCLV3* saplings. (a) Apical view of wild-type, *CRISPR-PagCLV3-2* and *CRISPR-PagCLV3-1-2* shoot meristems. (b) Apical view of shoot meristems examined by scanning electron microscope. The fasciation phenotype was more severe in *CRISPR-PagCLV3-1-2* than in *CRISPR-PagCLV3-2* lines. Red arrows indicate the band-shaped meristem. Bars = 100 μm.
