# Supplementary material for: The PagWUS-PagCLV3 module regulates shoot meristem maintenance and activity in poplar
Source: For Res (Fayettev). 2026 Mar 26;6:e007. doi: 10.48130/forres-0026-0007 (PMC13191361; doi:10.48130/forres-0026-0007)
Supplement: Supplementary file 1 — Supplementary data to this article can be found online. [file FR-2026-6-007-S1.zip › 10.48130_forres-0026-0007-Suppl-FigureS14.pdf]

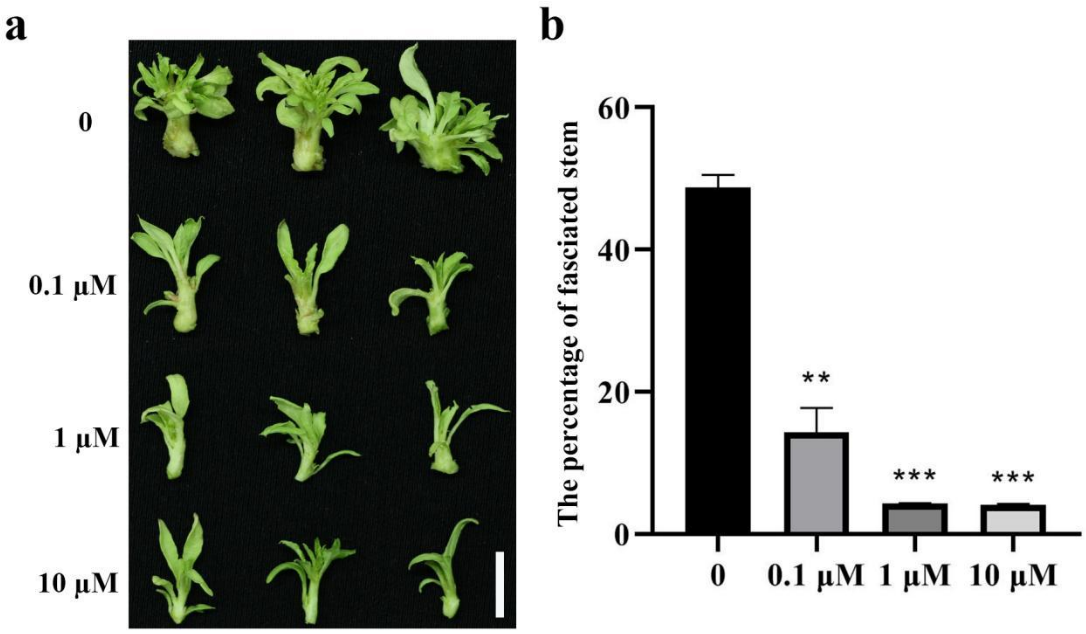

Supplementary Fig. S14

Exogenous PagCLV3-2 rescued the phenotype of *CRISPR-PagCLV3-2* shoots. (a) Phenotype of *CRISPR-PagCLV3-2* shoots exposed to different concentration of PagCLV3-2 peptide (indicated on left). (b) Statistic of the percentage of fasciated stem in (a). Bar = 1 cm. Data are mean ± s.d. of three independent biological repeats. **0.001 $<P < 0.01$ and ***$P < 0.001$ are determined by two-tailed Student's t-tests.
