# Supplementary material for: The PagWUS-PagCLV3 module regulates shoot meristem maintenance and activity in poplar
Source: For Res (Fayettev). 2026 Mar 26;6:e007. doi: 10.48130/forres-0026-0007 (PMC13191361; doi:10.48130/forres-0026-0007)
Supplement: Supplementary file 1 — Supplementary data to this article can be found online. [file FR-2026-6-007-S1.zip › 10.48130_forres-0026-0007-Suppl-FigureS15.pdf]

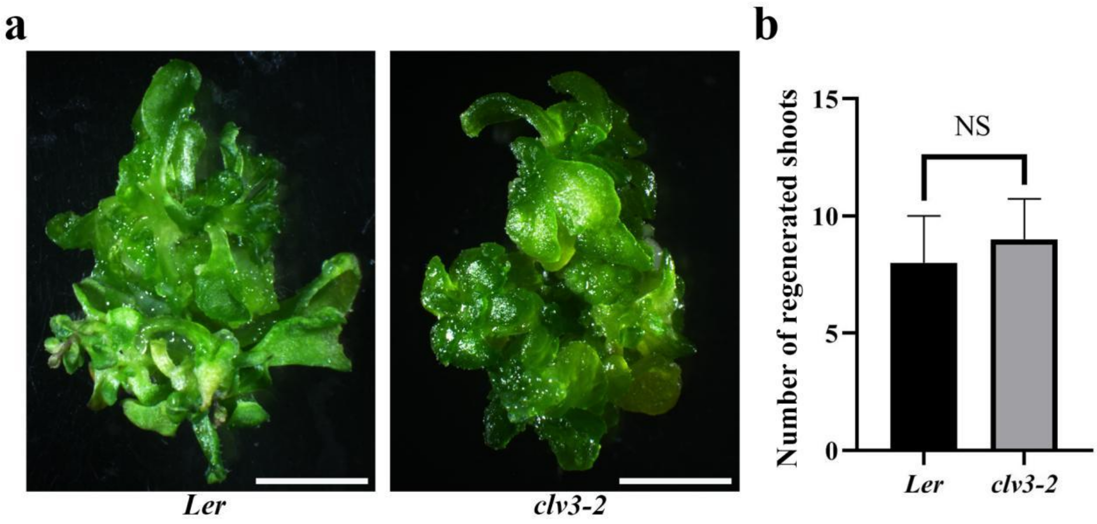

Supplementary Fig. S15

Shoot regeneration of Arabidopsis *clv3* mutant did not show obvious difference from the wild-type control. (a) Shoot regeneration of Arabidopsis *clv3* leaf explant was indistinguishable from that of wild type. (b) Numbers of regenerated shoots per leaf explant did not show significant difference between wild type and *clv3* mutant. Bar = 5 mm. Data are mean ± s.d. of three independent biological repeats. NS (No Significance) $P > 0.05$ are determined by two-tailed Student's t-tests.
