# Supplementary material for: The PagWUS-PagCLV3 module regulates shoot meristem maintenance and activity in poplar
Source: For Res (Fayettev). 2026 Mar 26;6:e007. doi: 10.48130/forres-0026-0007 (PMC13191361; doi:10.48130/forres-0026-0007)
Supplement: Supplementary file 1 — Supplementary data to this article can be found online. [file FR-2026-6-007-S1.zip › 10.48130_forres-0026-0007-Suppl-FigureS16.pdf]

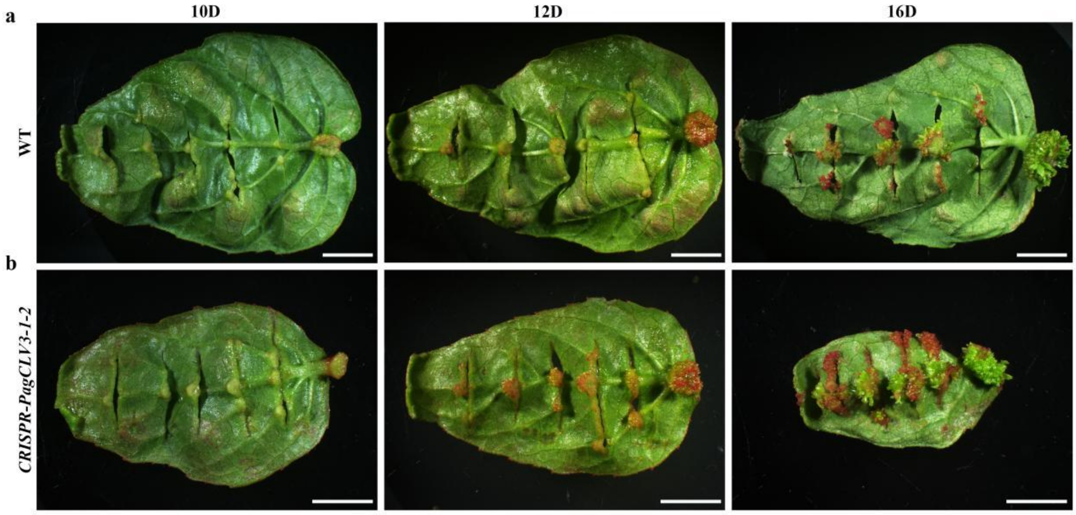

Supplementary Fig. S16

Shoot regeneration of wild-type and *CRISPR-PagCLV3-1-2* explants. Wild-type (a) and *CRISPR-PagCLV3-1-2* (b) explants at different stages of shoot regeneration, which was used for qRT-PCR analysis in Figure 5d. D indicates days after incubation. Bars = 2 mm.
