# Supplementary material for: The PagWUS-PagCLV3 module regulates shoot meristem maintenance and activity in poplar
Source: For Res (Fayettev). 2026 Mar 26;6:e007. doi: 10.48130/forres-0026-0007 (PMC13191361; doi:10.48130/forres-0026-0007)
Supplement: Supplementary file 1 — Supplementary data to this article can be found online. [file FR-2026-6-007-S1.zip › 10.48130_forres-0026-0007-Suppl-FigureS17.pdf]

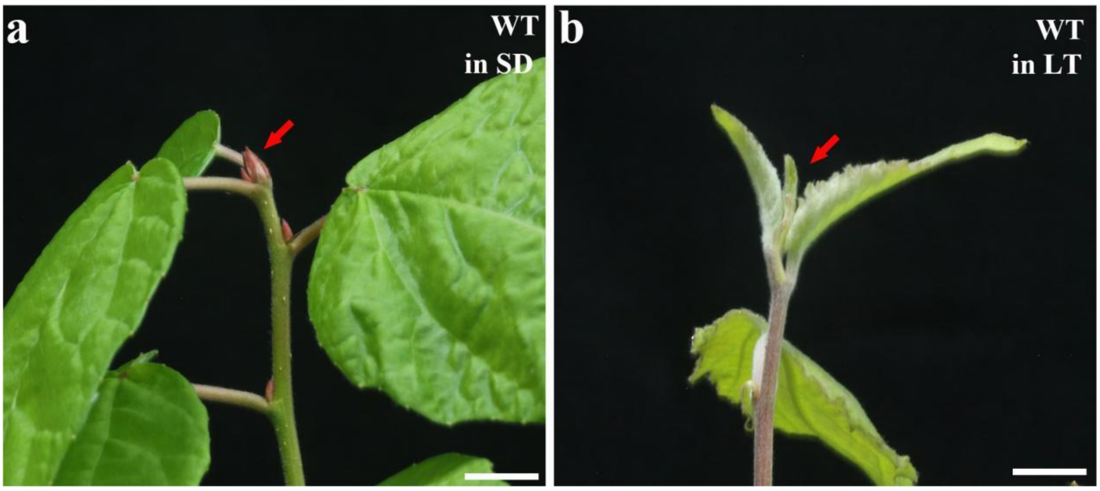

Supplementary Fig. S17

Shoot meristem activity was controlled by day-length. (a) Seven weeks short-day treatment led to growth cease of wild-type shoot tip. (b) Wild-type shoot tip grew normally after 7 weeks low-temperature treatment. Red arrows indicate shoot meristem. Bar = 1 cm.
