# Supplementary material for: The PagWUS-PagCLV3 module regulates shoot meristem maintenance and activity in poplar
Source: For Res (Fayettev). 2026 Mar 26;6:e007. doi: 10.48130/forres-0026-0007 (PMC13191361; doi:10.48130/forres-0026-0007)
Supplement: Supplementary file 1 — Supplementary data to this article can be found online. [file FR-2026-6-007-S1.zip › 10.48130_forres-0026-0007-Suppl-FigureS18.pdf]

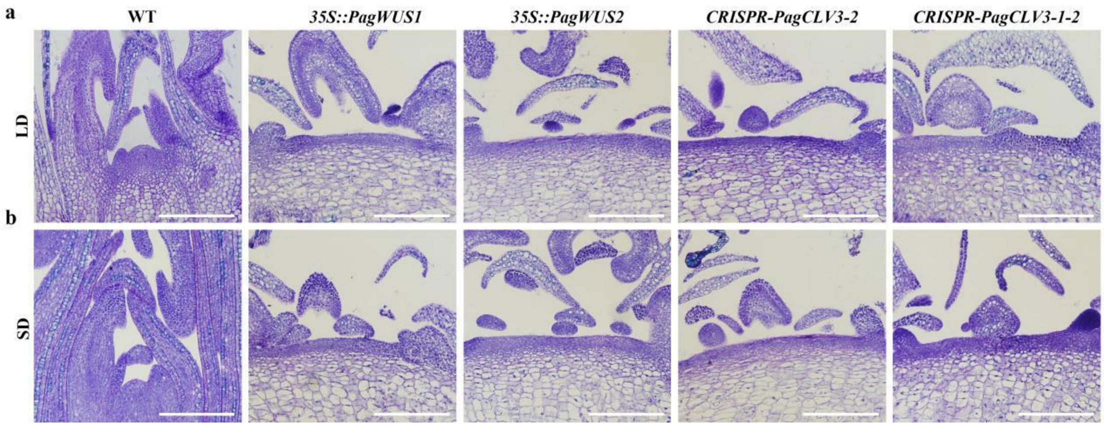

Supplementary Fig. S18

PagWUS-PagCLV3 module is involved in regulating shoot meristem cessation. Histological analysis of wild-type, *35S::PagWUS1*, *35S::PagWUS2*, *CRISPR-PagCLV3-2* and *CRISPR-PagCLV3-1-2* shoot meristems under long-day (a) and short-day (b) conditions for 4 weeks. Bars = 200 µm.
