# Supplementary material for: The PagWUS-PagCLV3 module regulates shoot meristem maintenance and activity in poplar
Source: For Res (Fayettev). 2026 Mar 26;6:e007. doi: 10.48130/forres-0026-0007 (PMC13191361; doi:10.48130/forres-0026-0007)
Supplement: Supplementary file 1 — Supplementary data to this article can be found online. [file FR-2026-6-007-S1.zip › 10.48130_forres-0026-0007-Suppl-FigureS19.pdf]

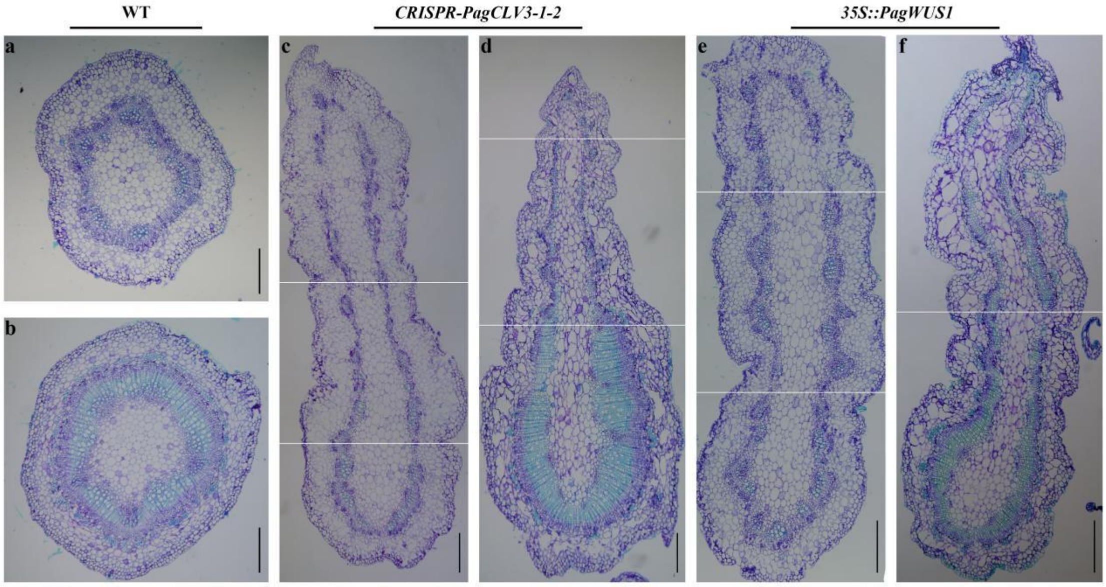

Supplementary Fig. S19

The *PagWUS-PagCLV3* module mediated proper secondary growth of the stem. Transection of the third (a) and tenth internode (b) of wild-type showed normal secondary growth pattern. In the upper part of *CRISPR-PagCLV3-1-2* stem, vascular bundles were evenly distributed (c), while the thickening process was uneven in the lower part (d). Similarly, the *35S::PagWUS1* stem demonstrated evenly distributed vascular bundles in the upper part (e), and uneven thickening process in the lower part of stem (f). Bars = 200 µm.
