# Supplementary material for: The PagWUS-PagCLV3 module regulates shoot meristem maintenance and activity in poplar
Source: For Res (Fayettev). 2026 Mar 26;6:e007. doi: 10.48130/forres-0026-0007 (PMC13191361; doi:10.48130/forres-0026-0007)
Supplement: Supplementary file 1 — Supplementary data to this article can be found online. [file FR-2026-6-007-S1.zip › 10.48130_forres-0026-0007-Suppl-FigureS4.pdf]

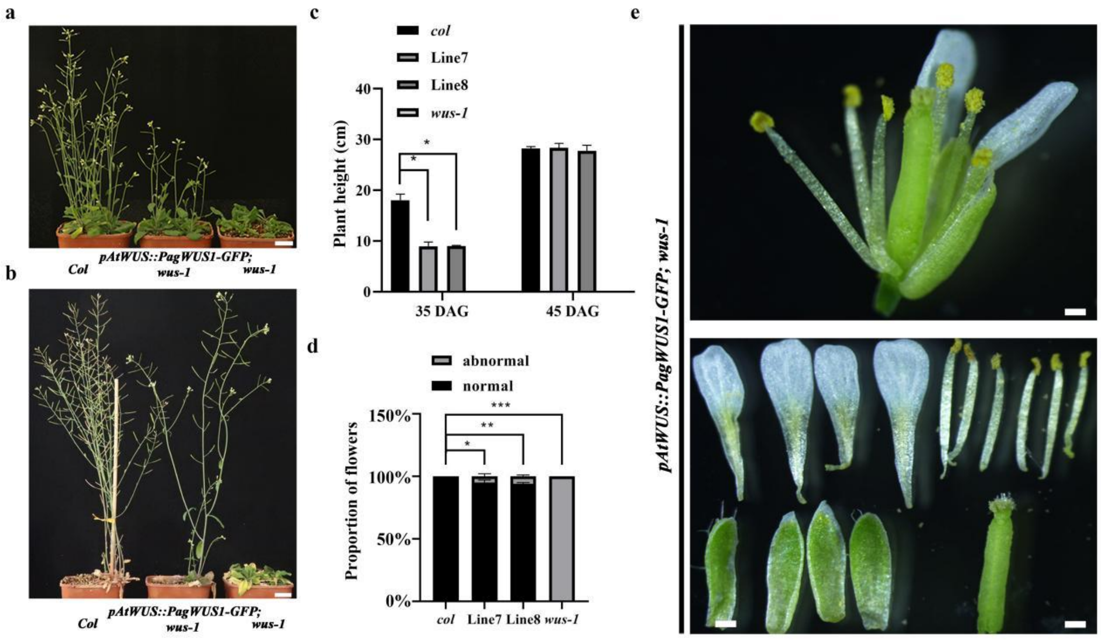

Supplementary Fig. S4

Expressing *pAtWUS::PagWUS1-GFP* largely rescues the phenotype of the *wus1* mutant. Comparisons among wild-type, *pAtWUS::PagWUS1-GFP* and *wus-1* seedlings at 35 days after germination (35 DAG) (a) and 45 DAG (b). (c) Statistics of plant height in (a) and (b). (d) Flower organ phenotype was near-complete rescued in the *pAtWUS::PagWUS1-GFP wus1* seedlings. (e) Flower derived from *pAtWUS::PagWUS1-GFP* seedling exhibited normal floral organs. n = 35. In total, 15 *pAtWUS::PagWUS1-GFP* lines with consistent and stable phenotypes were obtained. Line 7 was used in (a), (b) and (e). For (a) and (b), bar = 2 cm. For (c), bar = 2 mm. *$P < 0.05$, **$0.001 < P < 0.01$ and ***$P < 0.001$ are determined by two-tailed Student's t-tests.
