# Supplementary material for: The PagWUS-PagCLV3 module regulates shoot meristem maintenance and activity in poplar
Source: For Res (Fayettev). 2026 Mar 26;6:e007. doi: 10.48130/forres-0026-0007 (PMC13191361; doi:10.48130/forres-0026-0007)
Supplement: Supplementary file 1 — Supplementary data to this article can be found online. [file FR-2026-6-007-S1.zip › 10.48130_forres-0026-0007-Suppl-FigureS5.pdf]

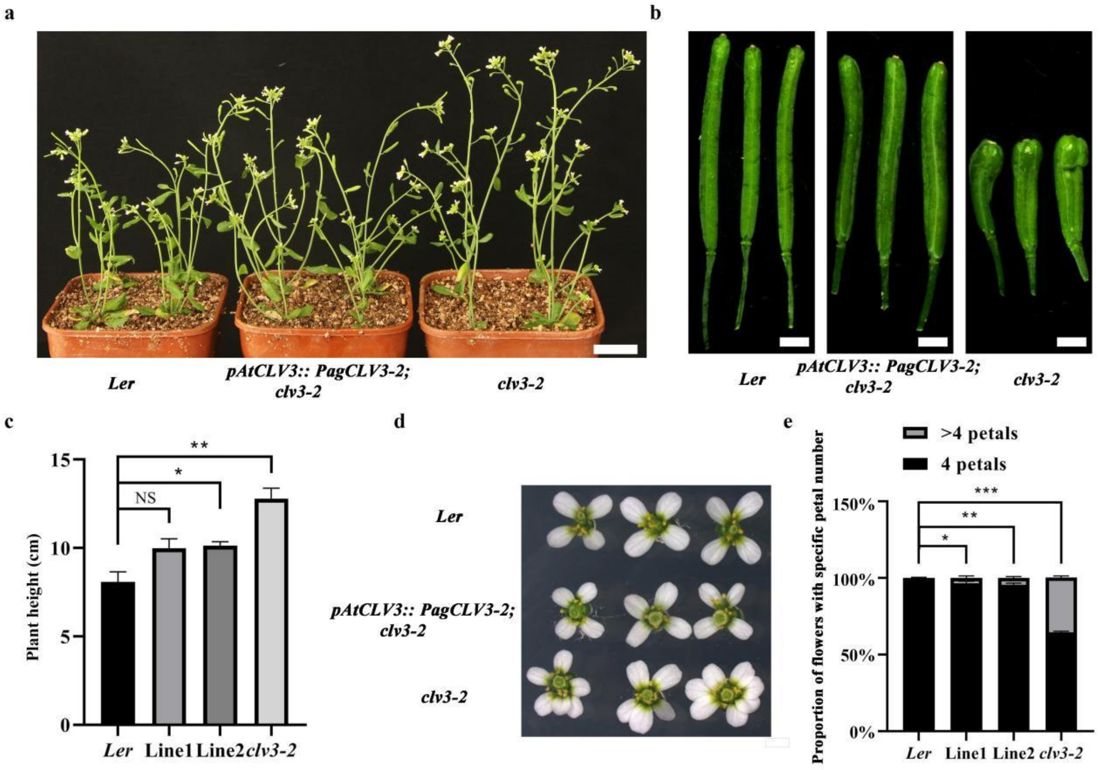

Supplementary Fig. S5

Introducing the *pAtCLV3::PagCLV3-GFP* vector partially rescues the *clv3-2* mutant phenotype. (a) Expressing *pAtCLV3::PagCLV3-GFP* partially rescued the *clv3-2* seedling phenotype at 30 DAG. For *pAtCLV3::PagCLV3-GFP clv3*, Line 1 seedlings are shown. Bar = 2 cm. (b) Silique phenotype of the *clv3-2* was rescued in the *pAtCLV3::PagCLV3-GFP clv3-2* lines. For *pAtCLV3::PagCLV3-GFP clv3*, siliques from Line 1 seedlings are shown. Bars = 2 mm. (c) Statistics of plant height in (a). (d) Flowers of the *pAtCLV3::PagCLV3-GFP clv3-2* lines were similar to those of the wild type. For *pAtCLV3::PagCLV3-GFP clv3*, flowers from Line 1 seedlings are shown. Bar = 2mm. (e) Proportion of petal numbers per flower of seedling of wild type (n = 15) , *clv3-2* mutant (n = 6) and two *pAtCLV3::PagCLV3-GFP clv3-2* lines (line1, n = 6; line2, n = 6). Data are mean ± s.d. of three independent biological repeats. In total, 12 *pAtCLV3::PagCLV3-GFP* lines with consistent and stable phenotypes were obtained. Line 1 was used in (a), (b) and (d). NS (No Significance), $P > 0.05$, $^{*}P < 0.05$, $^{**}0.001 < P < 0.01$ and $^{***}P < 0.001$ are determined by two-tailed Student's t-tests.
