# Supplementary material for: The PagWUS-PagCLV3 module regulates shoot meristem maintenance and activity in poplar
Source: For Res (Fayettev). 2026 Mar 26;6:e007. doi: 10.48130/forres-0026-0007 (PMC13191361; doi:10.48130/forres-0026-0007)
Supplement: Supplementary file 1 — Supplementary data to this article can be found online. [file FR-2026-6-007-S1.zip › 10.48130_forres-0026-0007-Suppl-FigureS6.pdf]

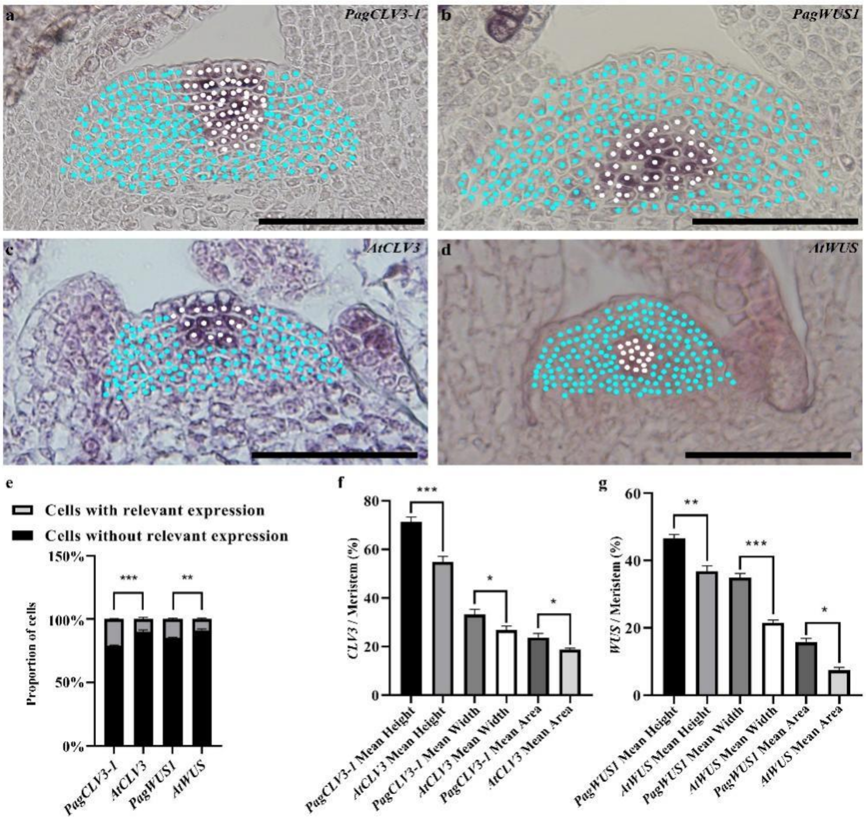

Supplementary Fig. S6

Proportion of cells constituting the stem cell niche in the total number of shoot meristem cell is larger in poplar than in Arabidopsis. (a-d) Cells constituting the stem cell niche and other meristematic cells in the shoot meristem of poplar (a and b) and Arabidopsis (c and d). White spots denote niche cells while bule spots mark meristematic cells out of the stem cell niche. Bar = 100μm. (e) Statistic data of (a-d). Percentages on each pillar represent the proportions of cells expressing relevant genes in the total meristematic cell number. (f) The ratios of height, width and area of the *PagCLV3*-expressing domain to those of the poplar meristem were higher than those of *AtCLV3*-expressing domain to the Arabidopsis meristem. (g) The ratios of height, width and area of the *PagWUS*-expressing domain to those of the poplar meristem were higher than those of *AtWUS*-expressing domain to the Arabidopsis meristem. For (a), n = 3; (b), n = 3; (c), n = 3; (d), n = 3. Data are mean ± s.d. of three independent biological repeats. *$P < 0.05$, **$0.001 < P < 0.01$ and ***$P < 0.001$ are determined by two-tailed Student's t-tests.
