# Supplementary material for: The PagWUS-PagCLV3 module regulates shoot meristem maintenance and activity in poplar
Source: For Res (Fayettev). 2026 Mar 26;6:e007. doi: 10.48130/forres-0026-0007 (PMC13191361; doi:10.48130/forres-0026-0007)
Supplement: Supplementary file 1 — Supplementary data to this article can be found online. [file FR-2026-6-007-S1.zip › 10.48130_forres-0026-0007-Suppl-FigureS7.pdf]

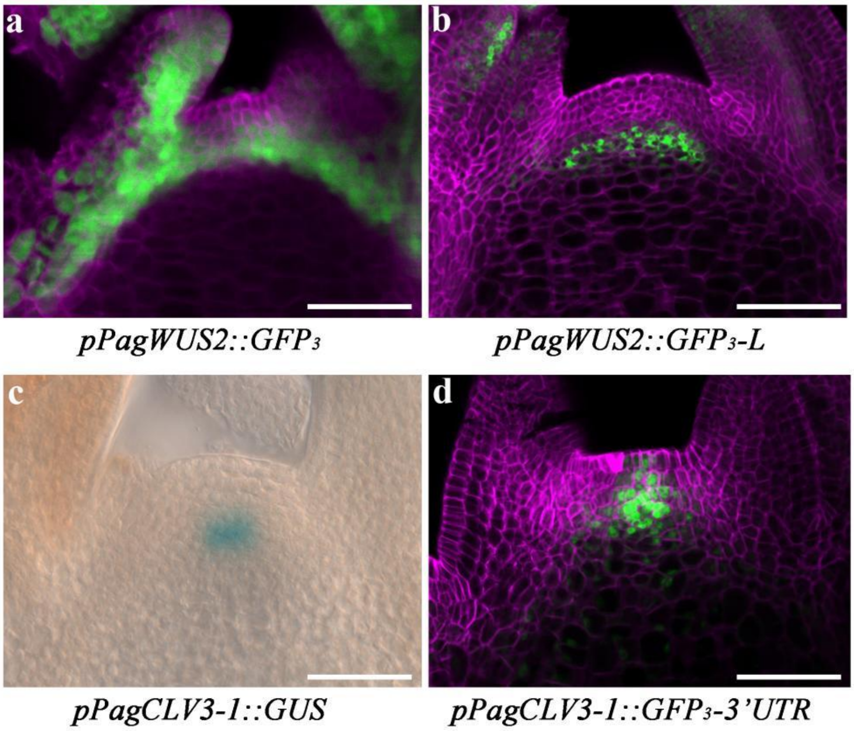

Supplementary Fig. S7

Expression patterns of reporters with different lengths of regulatory sequences. (a) When the 2408 bp sequence immediately upstream of the ATG start codon of *PagWUS2* was used as promoter, *PagWUS2::GFP$_3$*.GFP signals were distributed beyond the OC into the PZ and leaf primordium. (b) When the 6186 bp sequence immediately upstream of the ATG start codon of *PagWUS2* was used as promoter, *PagWUS2::GFP$_3$*-L signals were visible in the OC and PZ. (c) When the 4707 bp sequence immediately upstream of the ATG start codon of *PagCLV3-1* was used as promoter, *PagCLV3-1::GUS* signals were localized in the OC. (d) When the 4707 bp sequence immediately upstream of the ATG start codon and the1791 bp sequence downstream to the translational stop codon were used together as regulatory sequences, *PagCLV3-1::GFP$_3$*-L signals were mainly detected in the OC, with low level signals be visible in the CZ . Bars = 100 μm.
