# Supplementary material for: The PagWUS-PagCLV3 module regulates shoot meristem maintenance and activity in poplar
Source: For Res (Fayettev). 2026 Mar 26;6:e007. doi: 10.48130/forres-0026-0007 (PMC13191361; doi:10.48130/forres-0026-0007)
Supplement: Supplementary file 1 — Supplementary data to this article can be found online. [file FR-2026-6-007-S1.zip › 10.48130_forres-0026-0007-Suppl-FigureS8.pdf]

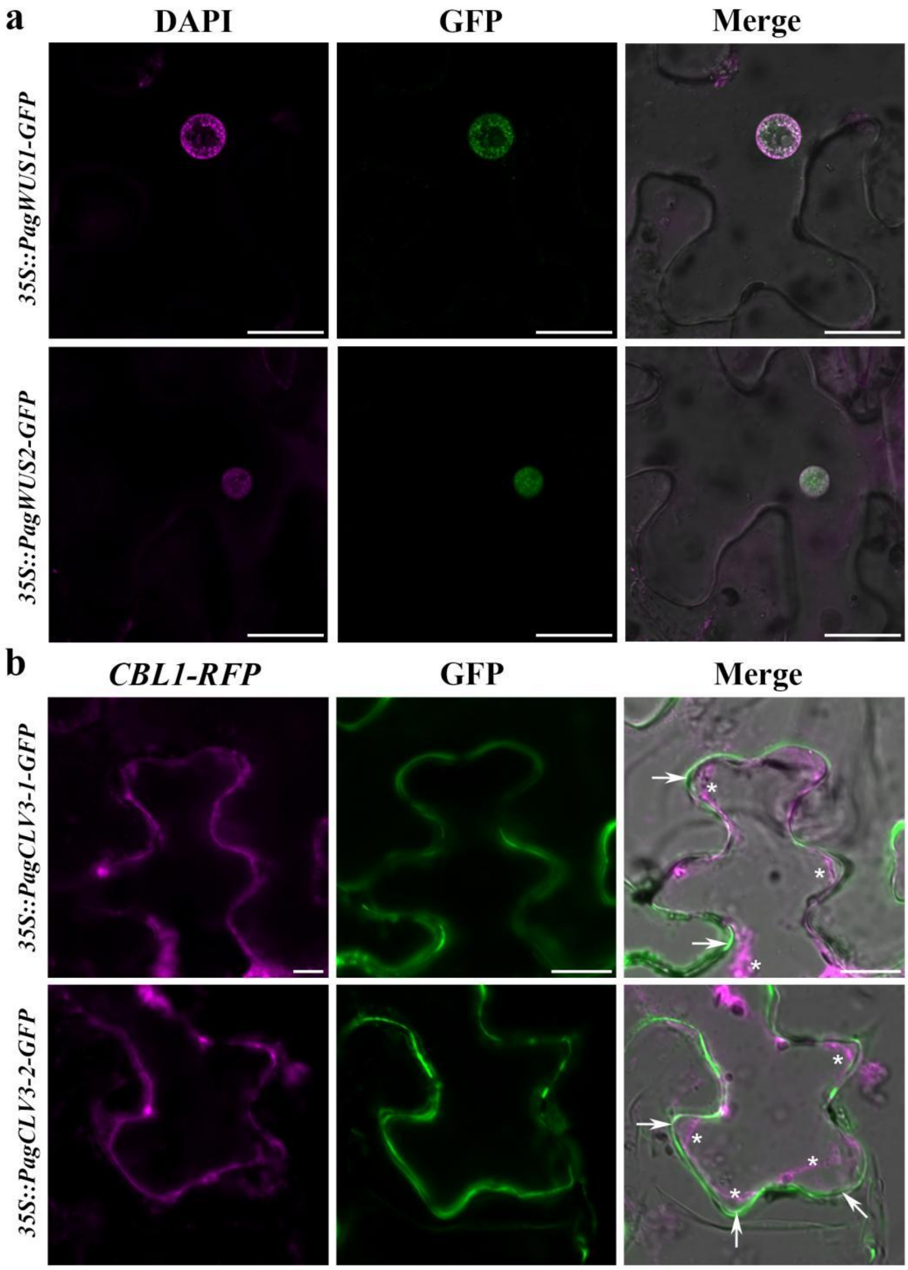


**Supplementary Fig. S8**

Subcellular localization of PagWUS1/2 and PagCLV3-1/2. (a) PagWUS1-GFP and PagWUS2-GFP signals were localized in the nucleus. (b) PagCLV3-1-GFP and PagCLV3-2-GFP signals were localized in the apoplast after plasmolysis. Plasma membrane is marked by *35S::CBL1-RFP*. Arrows indicate GFP signals in the apoplast. Asterisks shows plasma membrane after plasmolysis. Bars = 20 µm.
