# Supplementary material for: The PagWUS-PagCLV3 module regulates shoot meristem maintenance and activity in poplar
Source: For Res (Fayettev). 2026 Mar 26;6:e007. doi: 10.48130/forres-0026-0007 (PMC13191361; doi:10.48130/forres-0026-0007)
Supplement: Supplementary file 1 — Supplementary data to this article can be found online. [file FR-2026-6-007-S1.zip › 10.48130_forres-0026-0007-Suppl-FigureS9.pdf]

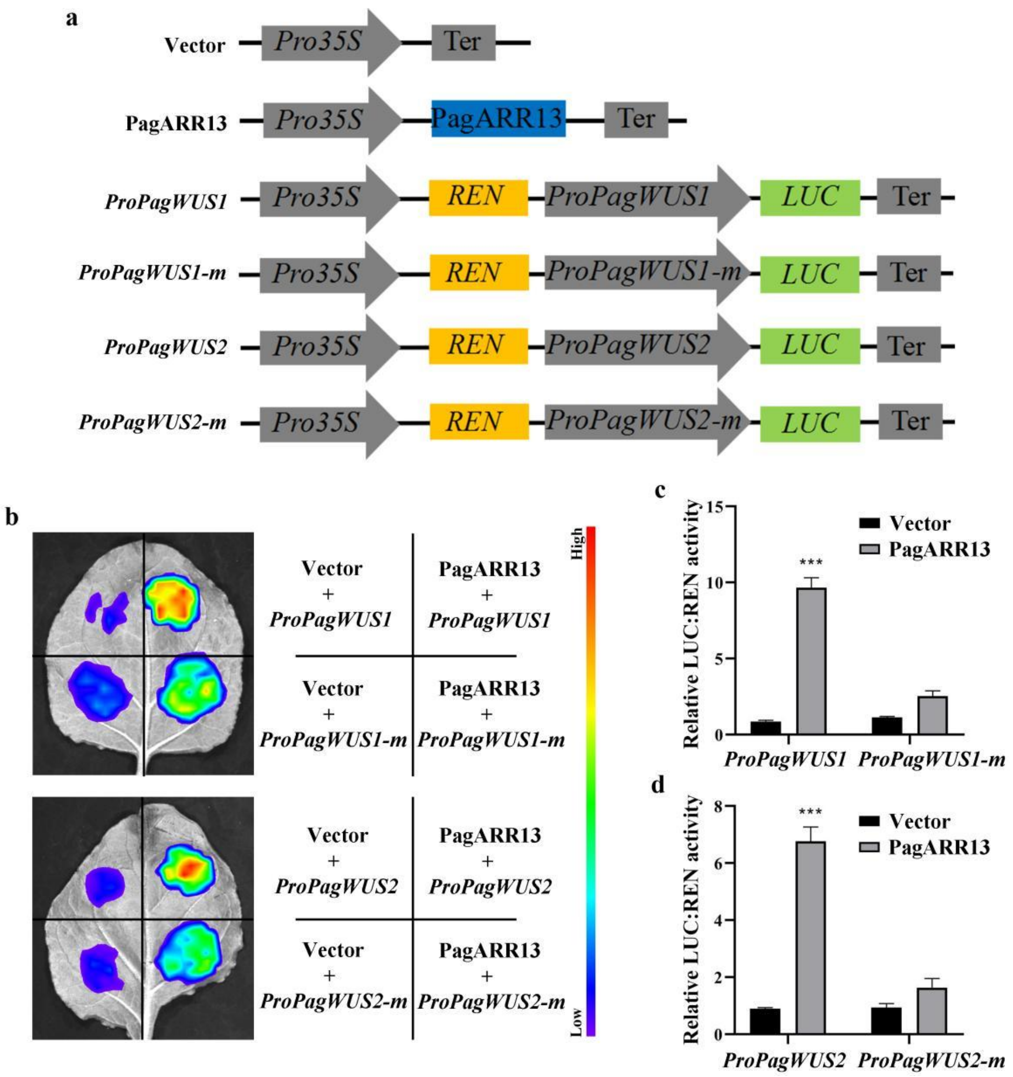

Supplementary Fig. S9

PagRR13 activates *PagWUS1/2* transcription. (a) Scheme of vectors used in the luciferase assay. *PagWUS1-m* indicates the mutation of GAT(T/C) to CTT(T/C) in *PagWUS1*-3 and *PagWUS1*-10 sequences, respectively. *PagWUS2-m* indicates the mutation of GAT(T/C) to CTT(T/C) in *PagWUS2*-1, *PagWUS2*-7 and *PagWUS2*-12 sequences, respectively. (b) Luciferase assay showing that PagRR13 activated the transcription of *PagWUS1/2* in tobacco leaves. The color bar indicates the intensity of luciferase activity. (c-d) Quantitative analysis of relative luciferase activity by expressing *ProPagWUS1:LUC* (c) and *ProPagWUS2:LUC* (d). Data are mean ± s.d. of three independent biological repeats. ***P < 0.001 are determined by two-tailed Student's t-tests.
