# Supplementary material for: The PagWUS-PagCLV3 module regulates shoot meristem maintenance and activity in poplar
Source: For Res (Fayettev). 2026 Mar 26;6:e007. doi: 10.48130/forres-0026-0007 (PMC13191361; doi:10.48130/forres-0026-0007)
Supplement: Supplementary file 1 — Supplementary data to this article can be found online. [file 10.48130_forres-0026-0007-Suppl-FigureS2.pdf › 10.48130_forres-0026-0007-Suppl-FigureS2.pdf]

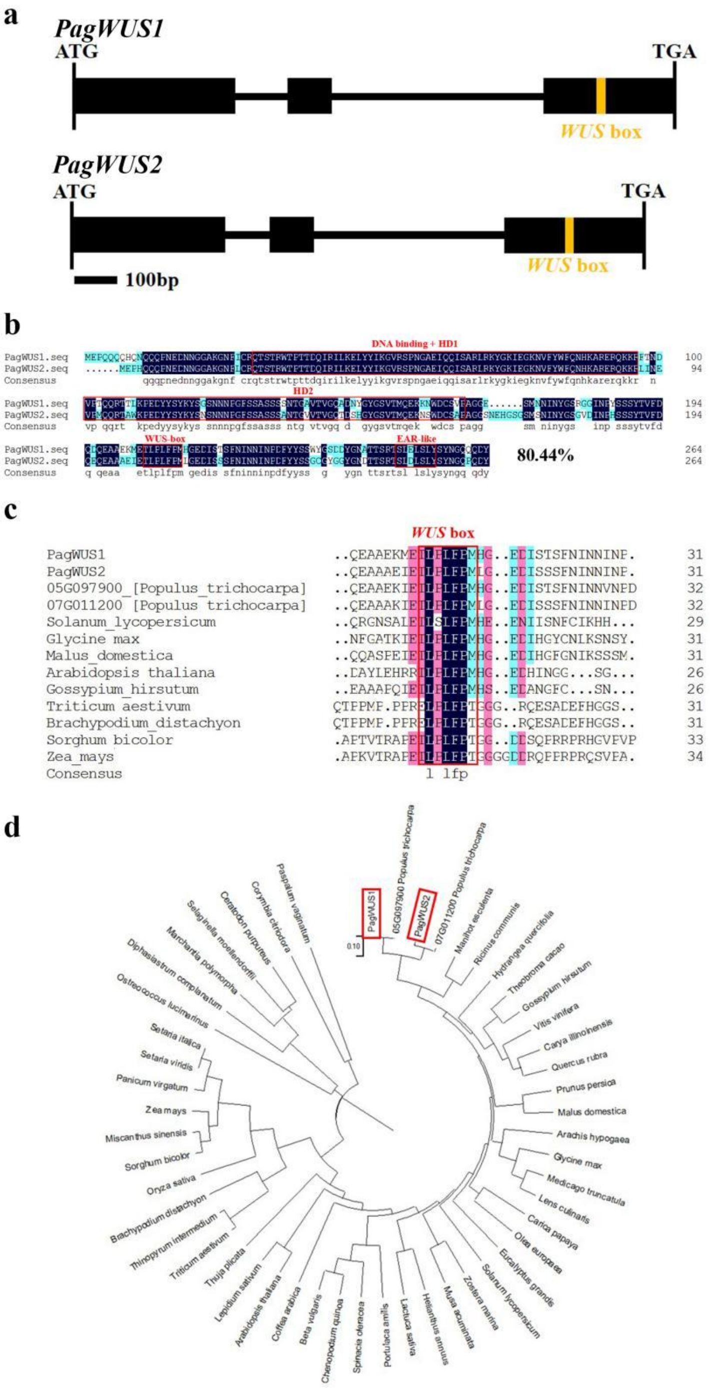

Supplementary Fig. S2

Structural and phylogenetic analysis of *PagWUS1* and *PagWUS2*. (a) Schematic diagram the conserved domains of *PagWUS1* and *PagWUS2*. (b) Similarity between amino acid sequences of PagWUS1 and PagWUS2. (c) Homology of *WUS* orthologs from different species. (d) Phylogenetic tree of the protein sequences of *WUS* orthologs from 49 different species.
